# Identification of unique highly hetero-substituted benzenes as chemical weapons of springtails by a combination of trace analytical methods with DFT calculations and synthesis†

**DOI:** 10.1039/d4sc03182b

**Published:** 2024-09-02

**Authors:** Anton Möllerke, Matthew Stell, Christian Schlawis, Ute Trauer-Kizilelma, Jennifer Herrmann, Hans Petter Leinaas, Stefan Scheu, Stefan Schulz

**Affiliations:** a Technische Universität Braunschweig, Institute of Organic Chemistry Hagenring 30 38106 Braunschweig Germany stefan.schulz@tu-braunschweig.de; b Umweltbundesamt Boetticherstraße 2 (Haus 19), Dahlemer Dreieck 14195 Berlin Germany; c Department of Microbial Natural Products, Helmholtz Institute for Pharmaceutical Research Saarland (HIPS) Campus E8.1 66123 Saarbrücken Germany; d Department of Bioscience, University of Oslo P. O. Box 1066 Blindern 0316 Oslo Norway; e University of Göttingen, JFB Institute of Zoology and Anthropology 37073 Göttingen Germany; f University of Göttingen, Centre of Biodiversity and Sustainable Land Use 37077 Göttingen Germany

## Abstract

Springtails (Collembola) are important members of the soil mesofauna. They are small, often less than 1–2 mm in length. A typical escape response of most surface-living species is to jump, using their furca. However, some species also use chemical defence against predators. While the defence chemistry of higher insects has been well studied, reports from the basal Collembola are rare, linked to the difficulties in obtaining enough biomass. We herein report on the identification and repellent activity of compounds detected in *Ceratophysella denticulata*. Extracts with various solvents obtained from only 50 individuals were sufficient for analysis by GC/MS, GC/HR-MS, and GC/IR. The large number of candidate structures of the major components were then prioritised by DFT calculations of IR spectra. Finally, the total synthesis of the top candidates confirmed the structures of the three major compounds to be 4-methoxy-5-(methylthio)benzo-1,3-dioxolane, 5,6,7-trimethoxybenzo-1,3-oxathiolane, and 8-amino-5,6,7-trimethoxybenzo-1,3-oxathiolane, the latter being the first naturally occurring fully hetero-substituted benzene. These highly substituted benzenes have no precedence in nature and carry structural motifs rare in nature, such as the benzo-1,3-oxathiolane ring system or the occurrence of O-, N-, and S-substituents at the same benzene core. Another novel natural compound, 2-methyl-1*H*-imidazo[4,5-*b*]pyridine, is used by *Hypogastrura viatica*. 4-Methoxy-5-(methylthio)benzo-1,3-dioxolane showed significant activity in deterrence assays with the ant *Lasius niger*. The data indicate that the title compounds are used in the chemical defence of these springtails, thus adding a new compound class to the known antipredator defences of arthropods. The results underline the difference in defence chemistry between Collembola and insects.

## Abstract 2

Tiny amounts form tiny springtails reveal highly hetereosubstituted benzenes as a new class of predator repellents.

## Graphical abstract

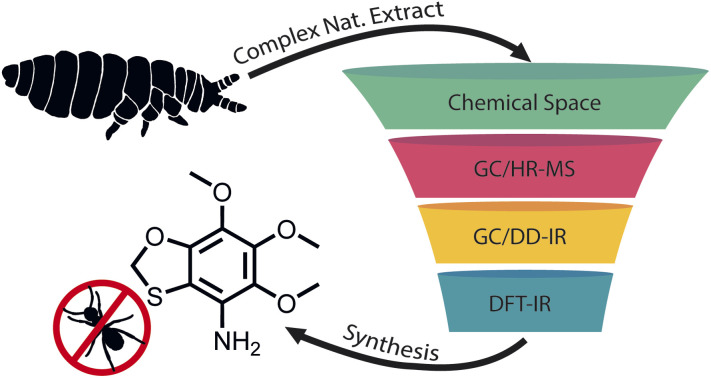

## Introduction

**Fig. 1:**
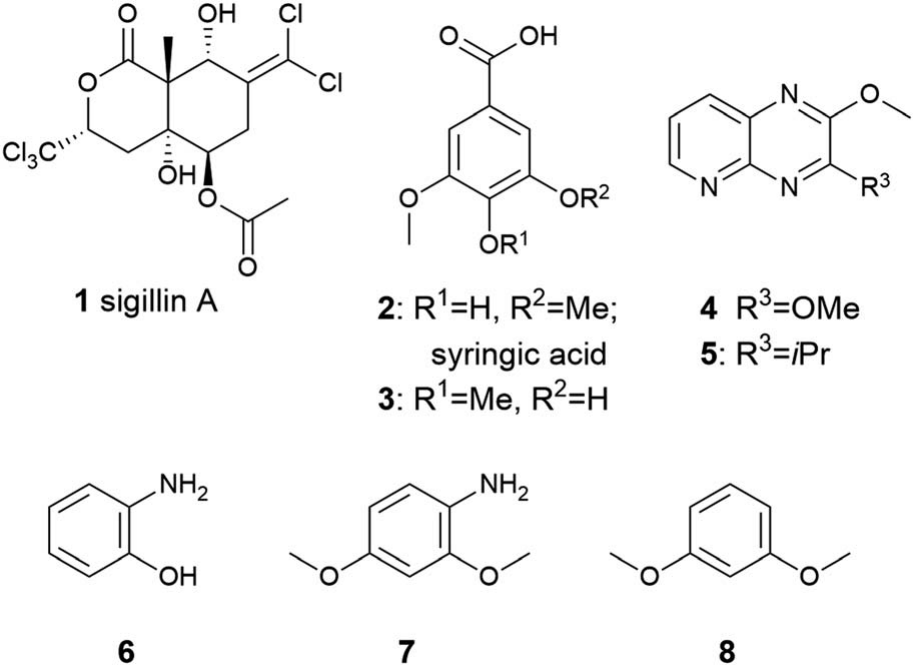
Compounds (1–8) associated with the chemical defence and signaling in Collembola.

Collembola as basal hexapods are important members of soil communities, living abundantly in ecosystems throughout the world. They diverged from other hexapods such as insects about 400 mya, explaining the often large differences found in secondary metabolites between these taxa. The usually tiny arthropods are also called springtails because they can move by catapulting themselves, *e.g.* to escape in dangerous situations, using their furca, a tail-like appendage. However, a number of species may also rely on chemical defence for protection (Fig. 1).

Examples include sigillins, such as sigillin A (1) from *Ceratophysella sigillata*,^1^ syringic acid (2), 3-hydroxy-4,5-dimethoxybenzoic acid (3) from *C. denticulata* (as *Hypogastrura denticulata*)^2^ or pyrido[2,3-*b*]pyrazines (4–5) from *Tetrodontophora bielanensis*,^3^*Onychiurus scotarius*, and *O. circulans*.^4^*Neanura muscorum* contained 2-aminophenol (6) and 2,4-dimethoxyaniline (7), with the former acting as a deterrent against the predatory mite *Pergamasus norvegicus*.^5^*N. muscorum* also uses the related 1,3-dimethoxybenzene (8) as an alarm pheromone.^6^

Only a few species of Collembola have been investigated so far for chemical defence, in contrast to insects. This might be due to the difficult sampling, their tiny size from only a few millimeters to sub-millimeter body length, and species determination requiring specialist expertise. The small arthropods are rarely collected in sufficient numbers in the wild to allow isolation of compounds, and cultivation in the laboratory is time-consuming and only achieved with a few species. Due to the trace amounts of analytes usually present in Collembola extracts, their analysis by GC/MS combines high sensitivity and good separation, and enables the analysis of samples from small animal numbers without further purification. The obtained mass spectra and gas chromatographic retention indices can be compared to large commercial databases such as NIST,^7^ and Wiley^8^ or smaller more specific ones such as MACE.^9^ This will allow easy identification of compounds such as 6, 7, or 8. However, while the interpretation of EI mass spectra can identify library unknowns according to well-established fragmentation rules,^10^ limitations still exist. These include the prediction of specific substitution patterns, stereochemical issues, functional group detection, or low-fragmenting groups such as arene systems. These issues can often only be resolved by comparison with synthetic material. We have previously shown that GC-coupled direct deposition infrared spectroscopy (GC/DD-IR) is a powerful addition to GC/MS-based structure elucidation, as it can handle the same complex mixtures with almost similar sensitivity, but provides additional information on functional groups.^11^ In addition, IR spectra can also be calculated using density functional theory (DFT).^12,13^

**Fig. 2:**
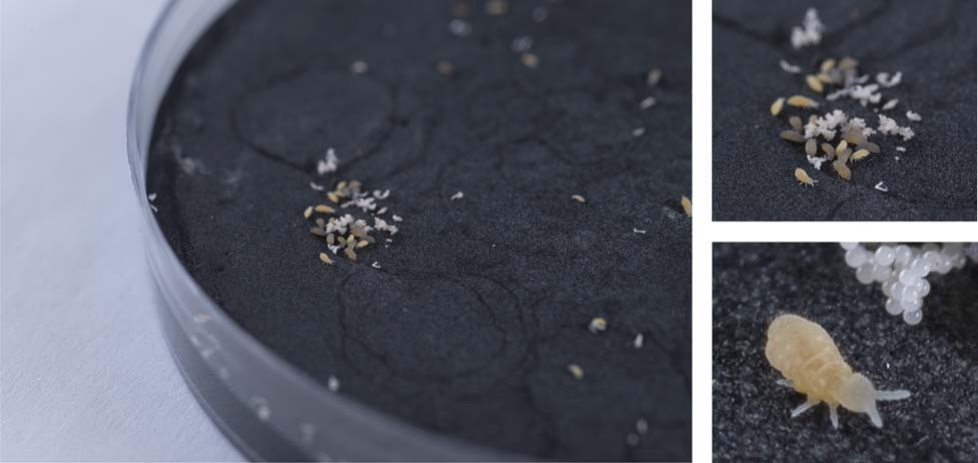
*Ceratophysella denticulata* on a Petri dish (*Φ* 9.4 cm) filled with plaster of Paris and activated charcoal.

Here we report the identification of a new class of natural products from *Ceratophysella denticulata* (Fig. 2) that are effective in antipredator defence. These compounds have a benzene core substituted by four to six heteroatoms, including the rare structural monothioacetal motif. To the best of our knowledge, highly to fully hetero-substituted benzenes have not yet been reported from nature. The structure elucidation was performed using samples from about 50 individuals, analysed by GC/MS, and GC/IR, supported by DFT calculations and final synthesis to prove structural proposals. The activity of the target compounds was tested in bioassays against insects and bacteria. Our work shows the effective cooperation of the described analytical techniques in the structural elucidation of trace amounts of analytes in complex mixtures without isolation or additional purification.

## Results

**Fig. 3:**
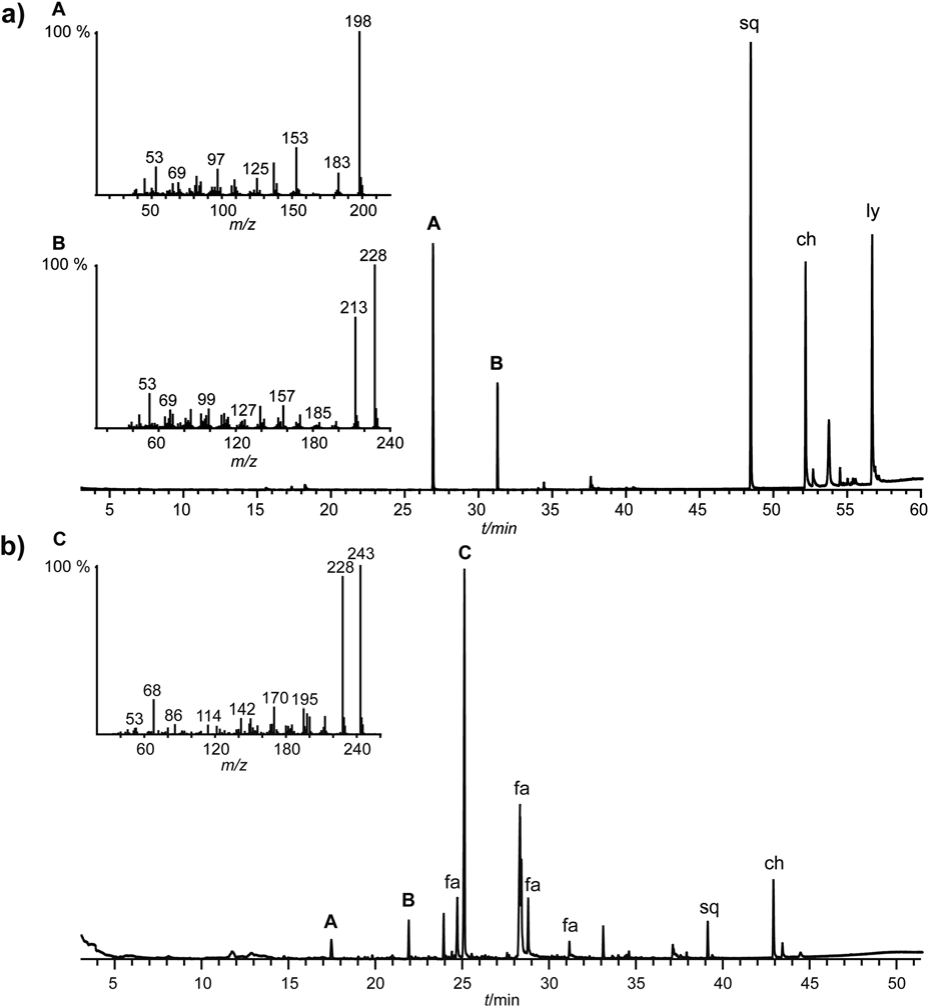
Total ion chromatograms of the pentane (a) and MeOH (b) extracts of *Ceratophysella denticulata*. The mass spectra of unknown compounds A, B, and C are shown. The separations were performed on an HP-5 phase. sq = squalene, ch = cholesterol, ly = lycopane, fa = fatty acid.

Individuals of *C. denticulata* were cultured in the laboratory, but their reproductive rate was slow, preventing the collection of sufficient numbers to isolate compounds in the quantities required for NMR analysis. Therefore, we opted for a GC-based approach. About 50 individuals (3–4 mg total) were extracted with pentane, followed by CH_2_Cl_2_ and MeOH. The total ion chromatogram (TIC) of the pentane extract (Fig. 3a) showed only a limited number of peaks, which is typical for Collembola extracts. This is in contrast to insect extracts which often contain complex mixtures of fatty acid-derived compounds. In addition to the typical terpenes squalene, lycopane, and cholesterol, we found two unknown compounds A (gas chromatographic retention *I* 1602) and B (*I* 1802).

### Structure elucidation

**Fig. 4:**
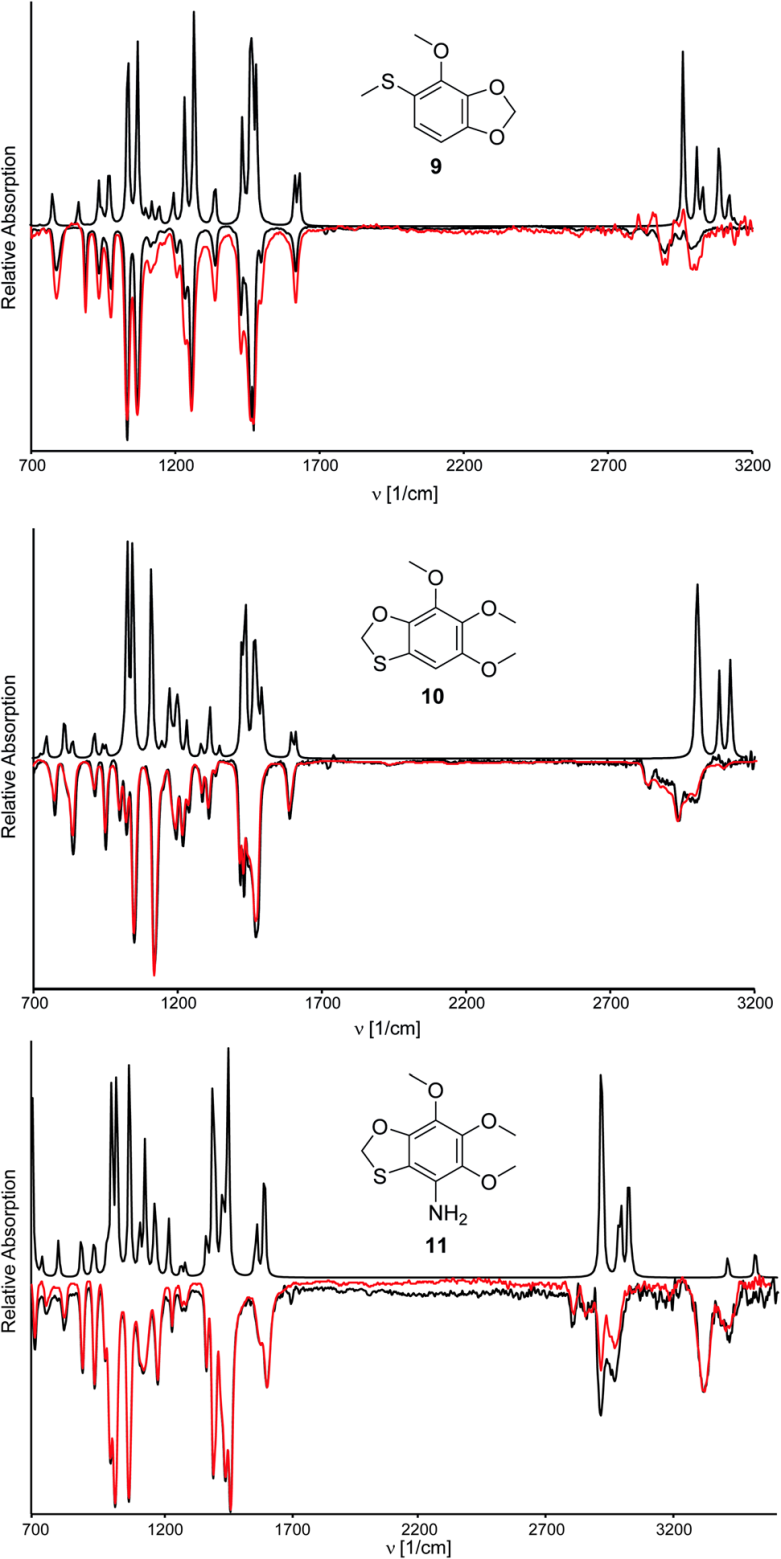
IR absorption spectra of natural compounds A, B, and C (negative, black), synthetic compounds 9, 10, and 11 (negative, red) and the DFT calculated spectra of 9, 10, and 11 (positive).

High-resolution MS indicated a molecular ion at *m*/*z* 198.03449 for compound A and *m*/*z* 228.04495 for compound B which correspond to the chemical formulae C_9_H_10_O_3_S (calc. 198.03452) and C_10_H_12_O_4_S (calc. 228.04508) respectively. In both cases, four double bond equivalents (DBE) were present, consistent with an aromatic ring. The intense M-15 ion indicated the presence of a cleavable methyl group. The CH_2_Cl_2_ extract showed no major difference compared to the pentane extract, other than small amounts of 1,3-dimethoxybenzene (8). However, the MeOH extract contained additionally an unknown compound C (*I* 1962) besides common fatty acids (Fig. 3b). High-resolution MS showed a molecular ion of *m*/*z* 243.05598 with the chemical formula C_10_H_13_NO_4_S (calc. 243.05598) for C. Interestingly, the aromatic acids 2 and 3, previously reported from this species,^2^ were not detected in the extracts. Although the mass spectral data seem to point to thiomethyl esters of 2 and 3 as potential structures for A and B, the IR data excluded this possibility. The IR spectra (Fig. 4) obtained by GC/DD-IR showed the absence of a carbonyl band. However, the spectra were consistent with aromatic compounds (2990 cm^−1^, br) containing an alkyl aryl ether or thioether (A: 1068 cm^−1^, s; 1033 cm^−1^, s; B: 1120 cm^−1^, s, 1050 cm^−1^, s).^14^ Interestingly, A showed a signal correlating to an aromatic dioxolane methylene (1256 cm^−1^, s)^14^ which is absent in B and C. Therefore, we proposed a benzodioxolane or benzoxathiolane core, with either one methoxy and one methyl sulfide substituent or two methoxy substituents for A and an additional methoxy substituent for B. Besides its similarities with B, C showed one unique additional feature, the signal at 3344 cm^−1^ (br) which indicated an arylamine, explaining the low solubility in apolar solvents. There are seven possible isomers for A and ten isomers for B with these characteristics, as well as ten plausible arylamines for C (Fig. S1†). We, therefore, needed to narrow down the field of candidates.

For prioritization, the IR spectra of all isomers were calculated by DFT methods (Fig. S2–S4†).^12^ The similarity between the spectra of the natural compound and the calculated ones is especially good in the fingerprint region. While visual inspection already prioritized three compounds, we also calculated the alignment of the experimental and calculated IR-spectra by the IRSA algorithm for an unbiased comparison (see ESI†).^13,15^ The highest similarity between the IR spectra in the fingerprint was between A and 9, between B and 10, and between C and **S16**† and 11 (Fig. 4 and ESI†). The visual inspection gave identical results, although 11 seemed to fit C slightly better than **S16**.† These results nevertheless needed verification by synthesis.

**Scheme 1:**
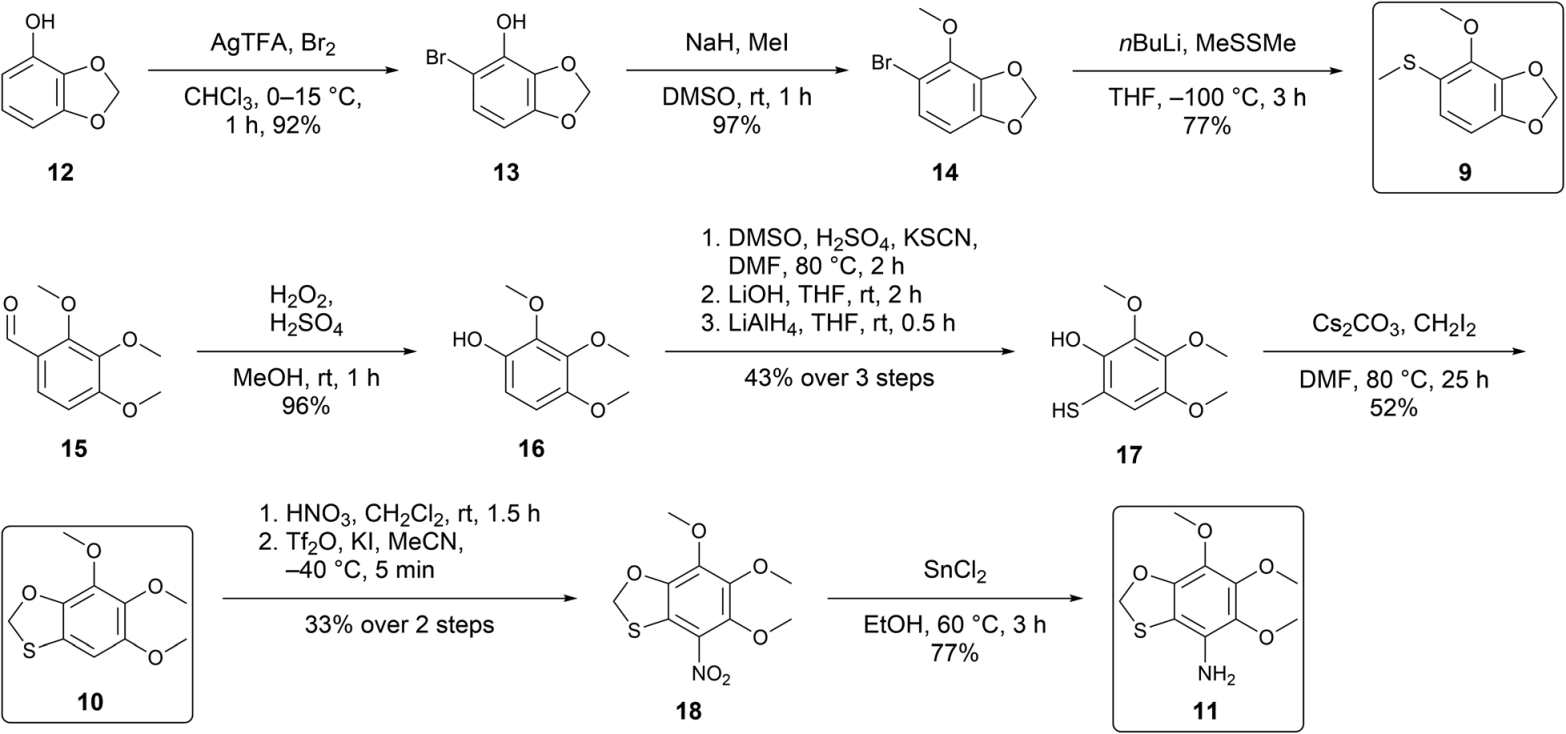
Synthesis of target compounds 9, 10, and 11.

The syntheses were performed to prove the structural proposals and to obtain material for bioassays to reveal their biological functions. For the synthesis of 9, bromination of 4-hydroxybenzo-1,3-dioxolane (12) (Scheme 1)^16^ furnished bromophenol 13 that was then methylated. A lithium halogen exchange and reaction with dimethyl disulfide followed, yielding the desired compound 9. Comparison of the retention index (A: *I* 1601, 9: *I* 1602), mass spectra (see ESI Fig. S5†), and IR data proved 4-methoxy-5-(methylthio)benzo-1,3-dioxolane (9) to be identical with the natural compound A.

A similar approach was used for the synthesis of 10 (Scheme 1). Aldehyde 15 was used as the starting material to afford phenol 16 through a Dakin oxidation.^17^ Phenol 16 is an electron-rich arene due to the numerous electron-donating groups, and to exploit this, we used an electrophilic aromatic substitution using DMSO as an oxidant.^18^ Mercaptophenol 17 was obtained after treatment with LiAlH_4_, followed by ring closure with diiodomethane to yield 10. Comparison of retention index (B: *I* 1801, 10: *I* 1802), mass spectra (Fig. S6†), and IR data proved 5,6,7-trimethoxybenzo-1,3-oxathiolane (10) to be identical with the natural compound B.

Nitration of 10 with HNO_3_ gave a mixture of 18 and the corresponding sulfoxide. Treatment of the crude mixture with Tf_2_O and KI ^19^ resulted in complete consumption of the sulfoxide but gave 18 only in low yields. The fully substituted benzene 18 was then reduced with SnCl_2_ in EtOH to obtain 11. Comparison of retention index (C: *I* 1962, 11: *I* 1962), mass spectrum (Fig. S7†), and IR data proved 8-amino-5,6,7-trimethoxybenzo-1,3-oxathiolane (11) to be identical with the natural compound C.

To verify, whether the good fit of calculated and measured IR-spectra also holds with other structures, 5,6-dimethoxy-4-(methylthio)benzo-1,3-dioxolane (**S12**†), the positional isomer of 10, was synthesized. The results showed again a very good fit of calculated and measured IR spectra, clearly different from compounds 9–11 (see ESI† for details).

Because substituted benzenes such as 1,3-dimethoxybenzene (8) have been reported from other springtails such as *Neanura muscorum*,^5,6^ we investigated other related species such as *Hypogastrura viatica* for the presence of these compounds. Though no hetero-substituted compounds were detected, we identified another aromatic compound, 2-methyl-1*H*-imidazo[4,5-*b*]pyridine (19) by analysis of its mass and IR spectra (Fig. S8†). This compound resembles the pyridopyrazines 4 and 5 previously identified as defence compounds of *Tetrodontophora bielanensis*,^3^ carrying a pyrazine ring instead of the imidazole ring.
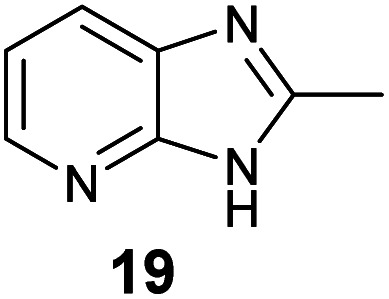


### Bioactivity assays

**Fig. 5:**
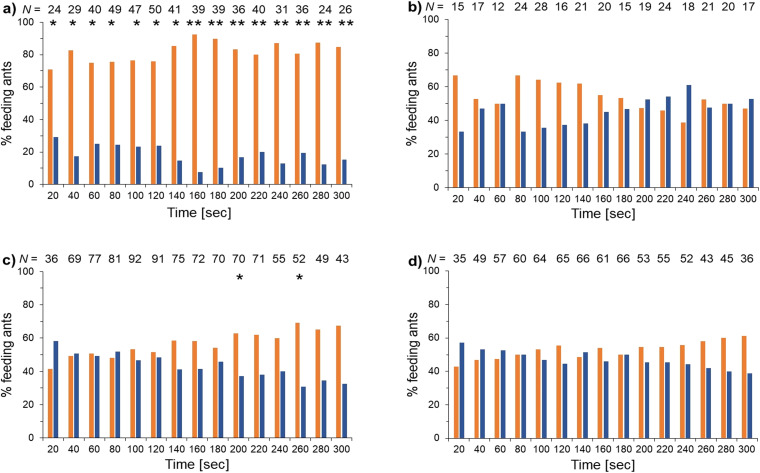
Feeding deterrence of compounds (a) 9, (b) 10, (c) 11, (d) 19 against the ant *Lasius niger* in a two-choice bioassay. Orange columns: percentage of ants feeding on 7 μL control solution (honey (40%) and condensed milk); blue columns: percentage of ants feeding on 7 μL test solution (0.1% w/w test compound in solution of honey (40%) and condensed milk). Test period 5 minutes. Every 20 seconds the number of ants feeding were counted. Statistical evaluation: Wilcoxon signed-rank test for paired differences; columns without asterisk showed no significant differences, *P < 0.05, **P < 0.01; numbers above the columns give the total number of ants feeding at this time. This bioassay was repeated 10 times for 9 and 10, 12 times for 11, and 13 times for 19.

Compounds 9, 10, 11, and 19 were then tested for their deterrence activity against the ant *Lasius niger* in a two-choice bioassay.^1,20–22^ This ant serves as a generalist predatory model insect that has been used in several bioassays for testing feeding deterrent activity in hexapods.^20,23^ Collembola are potential prey of *L*. *niger*,^24^ which forages both on soil and in vegetation for food. Artificial food consisting of honey and condensed milk was offered to the ants with and without the test compounds and their feeding response was detected (Fig. 5).

Compound 9 significantly deterred ants for the whole test period. In contrast, 10 showed no statistically significant effect. Some deterrence was observed with compound 11 which seems to increase over time, even so, the effect was only significant in two time points. Compound 19 showed no statistically significant repellency. The effectiveness of collembolan defence varies with its predator.^25^ Because we tested only one predator it might be possible that the compounds are active against other predators.

In addition, compounds 9, 10, 11, and 19 were tested in a panel of microorganisms for antibacterial as well as antifungal activities. No relevant inhibition was observed (Table S2†). The tested compound 9 therefore has a deterrent effect against a generalist predator but shows no antimicrobial activities. This is in line with the activities of other collembolan deterrents such as sigillins (1)^1^ or pyridopyrazines (4, 5)^3^ and unpublished results. Collembola lifestyles are closely associated with soil bacteria, both might be adapted to each other. Therefore, the production of highly active antimicrobial, secondary metabolites might be disadvantageous for Collembola, explaining their rarity.

The highly heterosubstituted benzenes 9, 10, and 11 constitute unique natural products, as fully or highly hetero-substituted benzenes were not reported from nature before. The benzooxathiolane and benzodioxolane cores contain methoxy, methyl sulfide, and/or amines, covering all major hetero elements important in biological systems. While benzo-1,3-dioxolanes are often encountered in nature, *e.g.* in lignins, benzo-1,3-oxathiolanes are extremely rare, an example being lissoclinidine B from the ascidian *Lissoclinum cf.* badium.^26^ Best to our knowledge, benzooxathiolanes or phenyl methyl sulfides have not been reported from arthropods before.

The oxidized benzoic acids 2 and 3 reported earlier from *C. denticulata* were absent in our samples.^2^ Compound 10 had been observed, although its structure remained unknown at that time.^27^ Still, 2 and 3 share a highly oxidized phenyl ring with our target compounds, suggesting potentially related biosynthetic pathways.

## Conclusion

In summary, we have established a protocol to identify minute amounts of secondary metabolites from springtails using sequential extraction followed by GC/MS, GC/HR-MS, and GC/DD-IR analysis. The evident structural elements delivered a set of likely candidate structures, which were prioritized by DFT calculations of IR spectra. As was proven by synthesis, this prioritization was correct. This confirmed our analytical protocol to be a valuable tool in structure elucidation, also delivering robust results for compounds previously unknown from nature, like benzoxathiolanes. The highly hetero-substituted compounds include the first fully hetero-substituted natural benzene, 11. Compounds 9 and 11 showed repellent activity against a generalist ant predator. These highly hetero-substituted benzenes compounds define a new class of feeding deterrents for arthropods.

## Author contributions

AM performed the cultivation, extraction, and analysis of the *C. denticulata*, as well as the synthesis. MS did the DFT calculations. CS did the preparation and analysis of *H. viatica*. UTK performed the two-choice feeding assay. JH tested the substances against the microorganism panel. HPL and SSscheu provided Collembola samples and biological background. SSchulz initiated the research and provided the strategy. AM and SSchulz wrote the draft manuscript. All authors contributed to the final manuscript.

## Conflicts of interest

There are no conflicts to declare.

## Supplementary Material

SC-015-D4SC03182B-s001

SC-015-D4SC03182B-s002

## Data Availability

The data supporting this article have been included as part of the ESI.† Mass spectral data for this article will be available after publication in the open access mass spectra repository MACE [http://www.oc.tu-bs.de/schulz/html/MACE.html],^1^ located at the Leopard server of our University. The current version mace_r5.1 at [https://doi.org/10.24355/dbbs.084-202402071310-0] will contain links to the appropriate website. IR data of the natural compounds and **S12**† can be found as ESI.†
