# Supplementary material for: Identification of unique highly hetero-substituted benzenes as chemical weapons of springtails by a combination of trace analytical methods with DFT calculations and synthesis
Source: Chem Sci. 2024 Sep 2;15(37):15332–8. doi: 10.1039/d4sc03182b (PMC11376146; doi:10.1039/d4sc03182b)
Supplement: SC-015-D4SC03182B-s001 [file SC-015-D4SC03182B-s001.pdf]

Supplementary Information (SI) for Chemical Science.
This journal is © The Royal Society of Chemistry 2024

Supporting Information for

Identification of unique highly hetero-substituted benzenes as chemical weapons of springtails by a combination of trace analytical methods with DFT calculations and synthesis

Anton Möllerke, Matthew Stell, Christian Schlawis, Ute Trauer-Kizilelma, Jennifer Herrmann, Hans Petter Leinaas, Stefan Scheu, Stefan Schulz*

* Corresponding author: stefan.schulz@tu-braunschweig.de

Content

DFT calculated IR Spectra	2
Mass Spectra	8
Antimicrobial Activity Test	10
Material and Methods	12
NMR Spectra	20
References	31

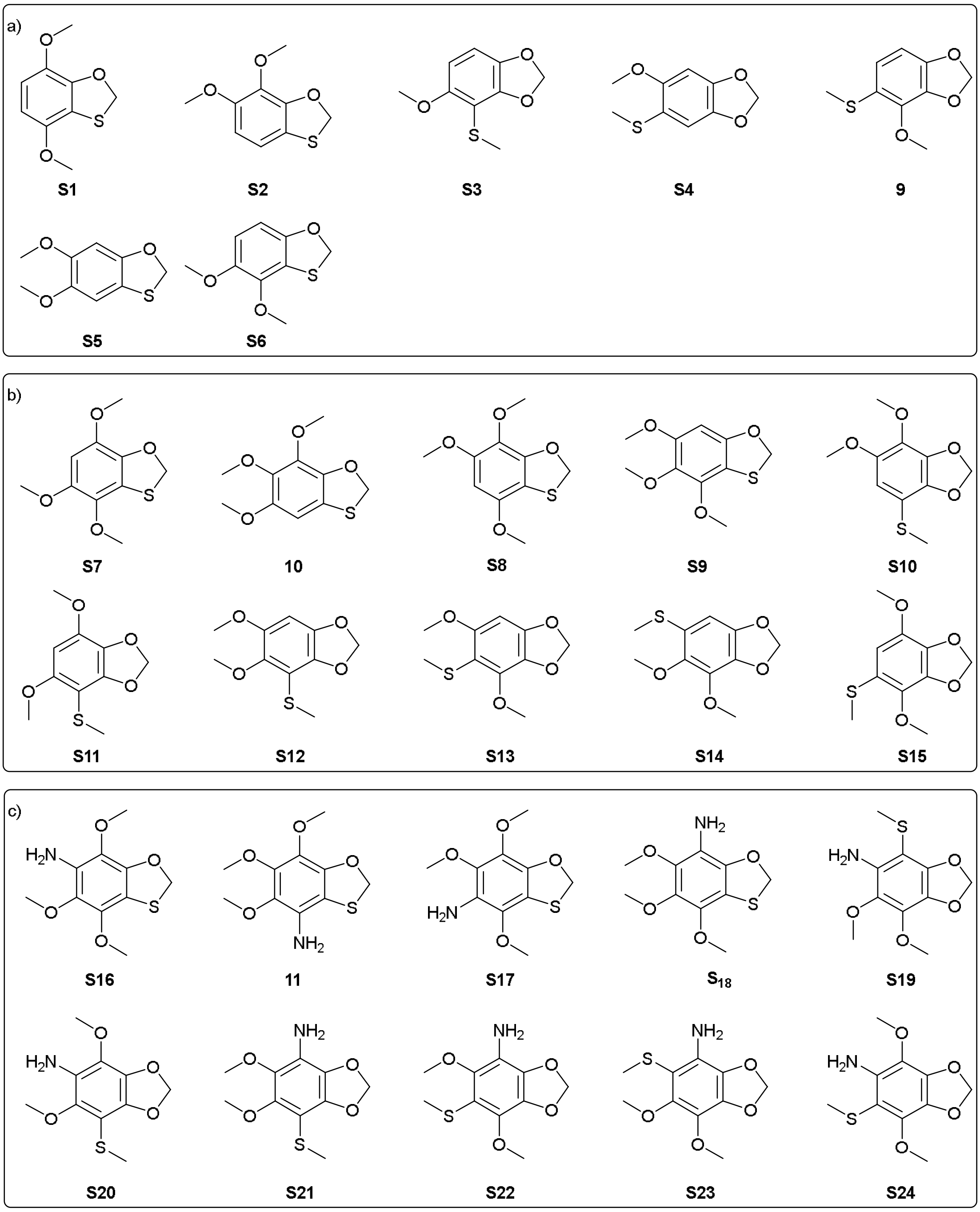

Figure S1. Scope of plausible structures of **A** (a), **B** (b), and **C** (c) for DFT-based IR calculations.

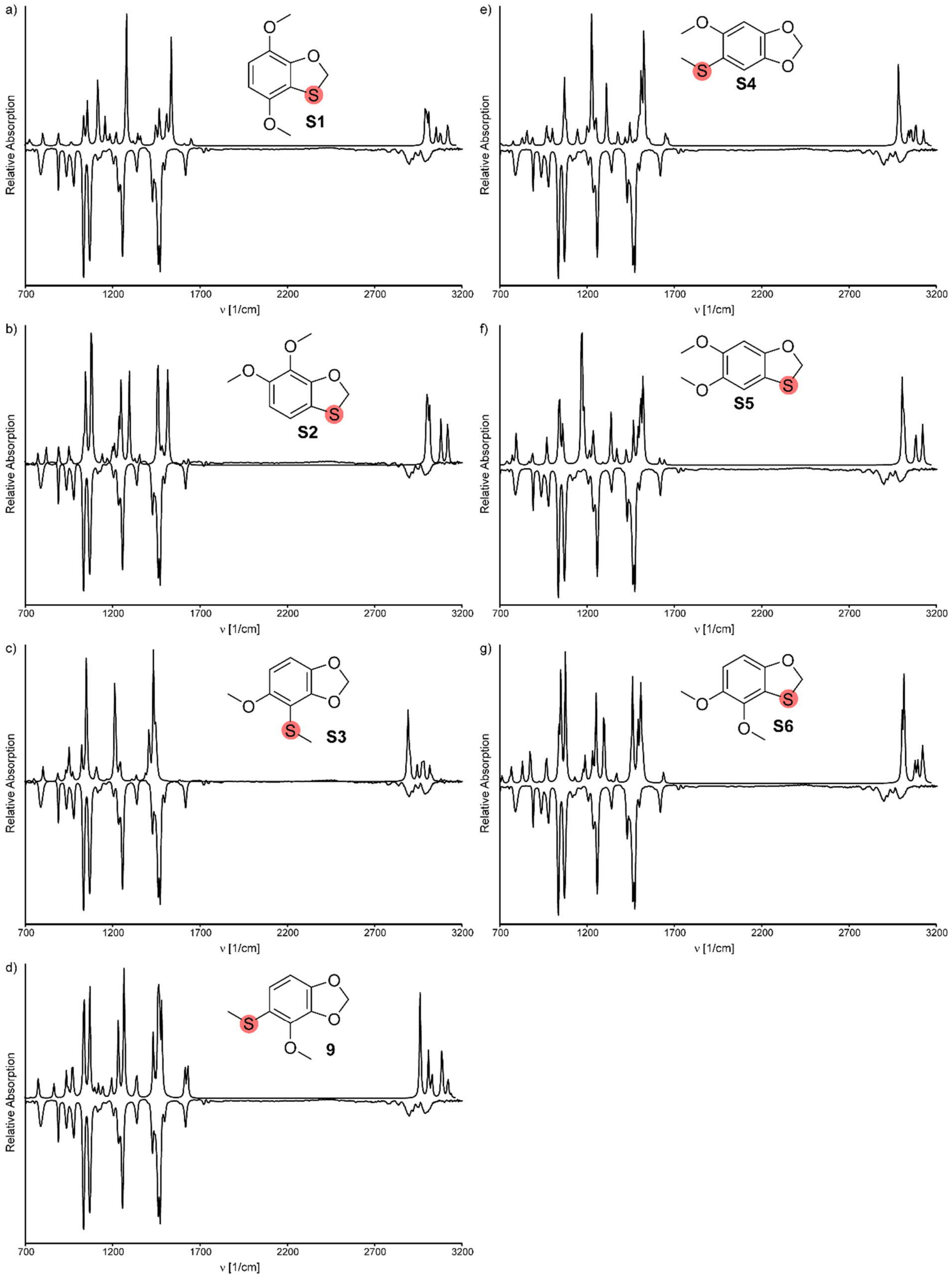

Figure S2. IR absorption of the natural compound **A** (negative) and the calculated absorption of **9** and **S1**–**S6** (positive).

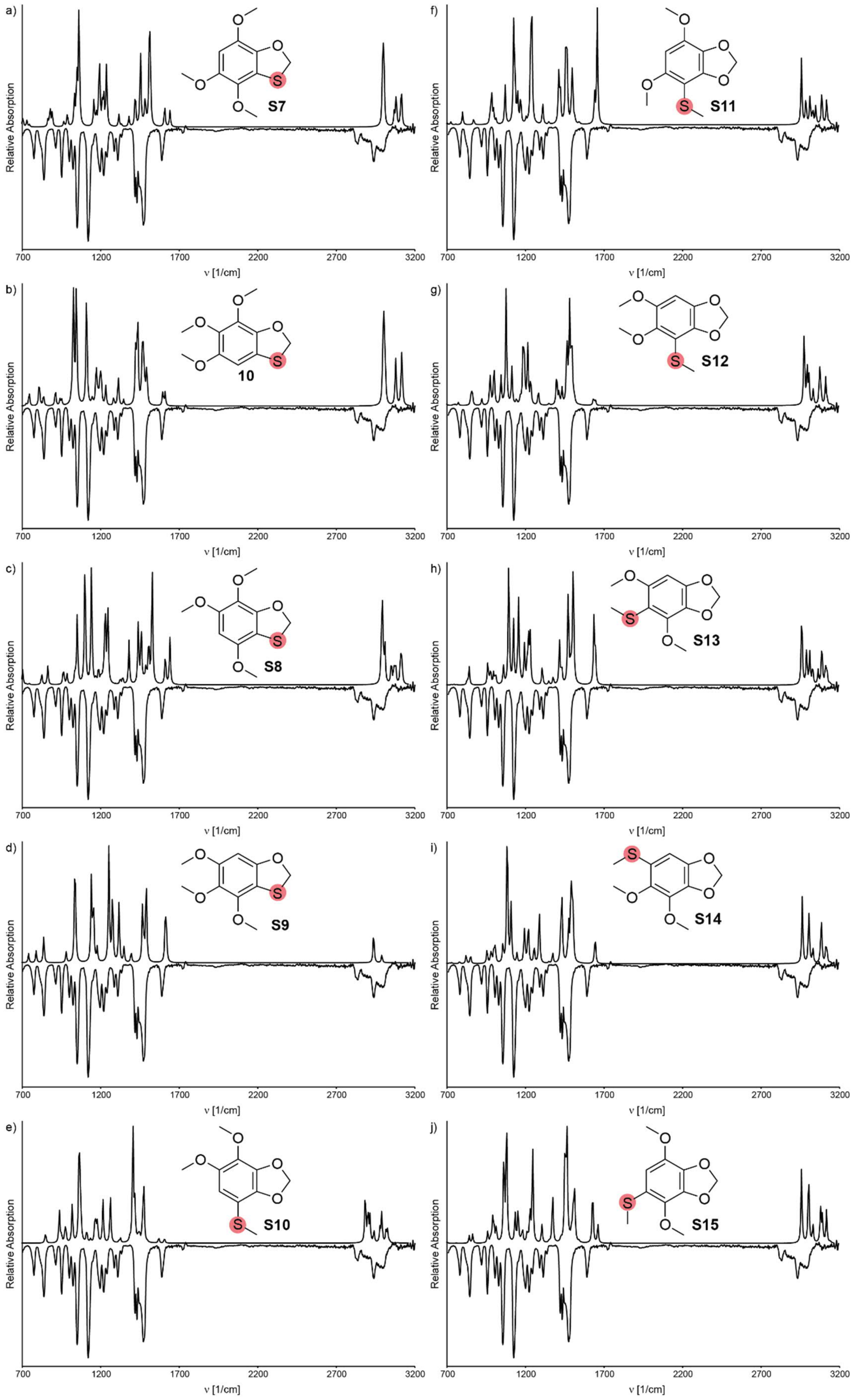

Figure S3. IR absorption of the natural compound **B** (negative) and the calculated absorption of **10** and **S7**–**15** (positive).

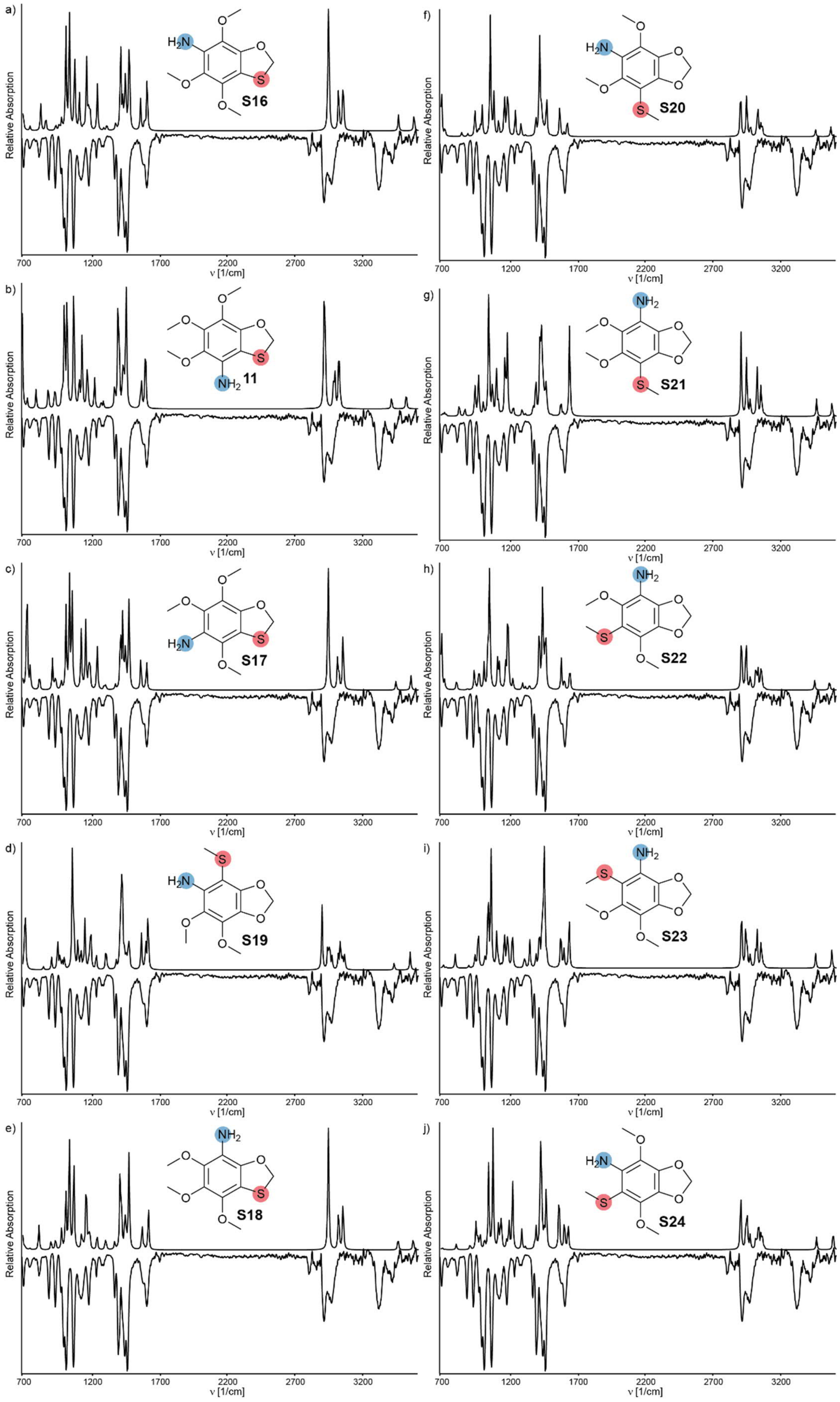

Figure S4. IR absorption of the natural compound **C** (negative) and the calculated absorption of **11** and **S16**–**24** (positive).

Comparison of DFT-generated IR data with the natural compounds

To quantify the alignment of the experimental and DFT-calculated IR spectra, we calculated the alignment using the IRSA algorithm for an unbiased comparison. The results were compared using Pearson's correlation coefficient (r_P).[1,2] The spectra show broad, not well-defined peaks in the region of 2800-3060 cm^{-1} (=C-H stretching and the N-H stretching bands in the case of the aromatic amines) with not well-fitting wrong wavenumbers and intensities. We therefore focused on the fingerprint region of 700-1700 cm^{-1}, which shows well-defined peaks. This fingerprint region is often compound-specific and has the highest value in identifying a particular substance. The peaks of the fingerprint region were manually selected in the experimental and calculated spectra. The IRSA scoring function was used:[1,2]

$$s_{i,j} = e^{-0.5(-1)^2/\sigma_1^2} \cdot e^{-0.5\left(\frac{\nu_i}{\nu_j}-\mu\right)^2/\sigma_2^2}$$

where s is the score that matches the experimental peak i with the theoretical peak j. I_i and I_j are the intensities of the experimental and theoretical intensities, respectively, and ν_i and ν_j are their respective wavenumbers. A constant scaling factor is μ, set to 1.00, and σ_1 = 0.1 and σ_2 = 0.0192 are parameters. The Pearson coefficient was calculated for each structure, the results are shown in Table S1.

The Pearson coefficient can have values between -1 and 1, where -1 means negative correlation, 0 means no correlation, and 1 perfect correlation. The best results in the comparisons of A and B were structures 9 and 10. The best result for C is S16 followed by 11. The respective r_P of both structures were highly similar. A visual inspection of both spectra favored compound 11. Because this compound was easily accessible from 10, we decided to synthesize 11 first.

Table S1. Pearson coefficient (r_P) describing the alignment of the calculated spectra with the experimental spectra of A, B, and C.

	S1	S2	S3	9	S4	S5	S6
r_P (A)	-0.198	0.012	-0.011	0.666	-0.084	-0.096	0. 491

	S7	10	S8	S9	S10	S11	S12	S13	S14	S15
r_P (B)	0.066	0.258	0.203	0.137	-0.182	0.002	-0.098	0.058	-0.141	-0.193
r_P (S12)	-0.079	-0.197	-0.181	-0.041	-0.041	-0.154	0.357	0.036	-0.097	0.014

	S16	11	S17	S18	S19	S20	S21	S22	S23	S24
r_P (C)	0.251	0.229	-0.078	0.109	-0.216	-0.124	-0.133	-0.124	-0.218	0.083

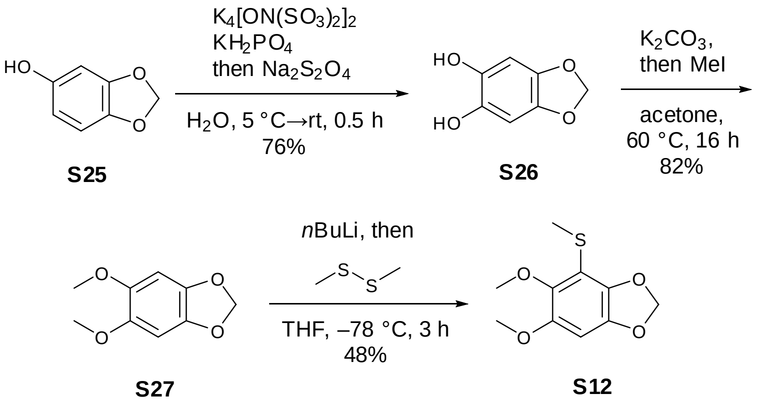

Scheme S1. Synthesis of S12 starting from sesamol (S25).

To verify our computational approach, we synthesized an additional isomer (Scheme S1), which is not found in the natural extracts. For the synthesis of S12 sesamol was oxidized to the corresponding *o*-hydroquinone (S27). Methylation followed by Li-H-exchange and quenching with dimethylsulfide (DMDS) gave S12.

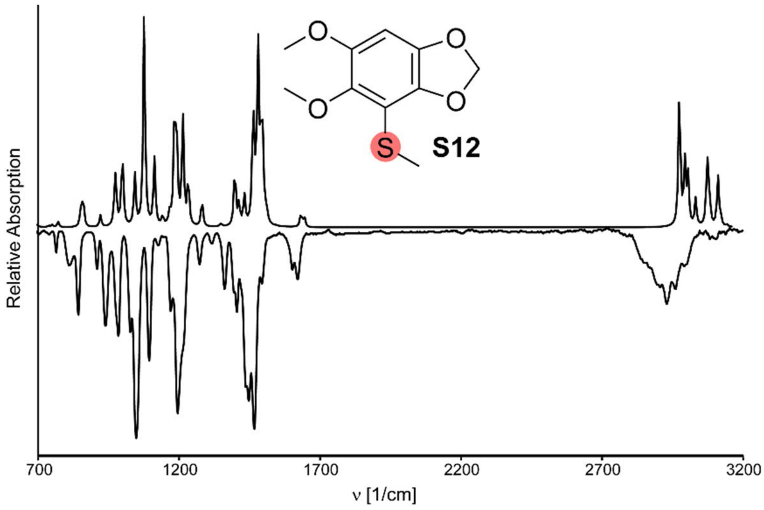

Figure S5. IR absorption spectra of the synthetic compound S12 (negative) and the spectra of the corresponding DFT-calculations (positive).

Mass spectra

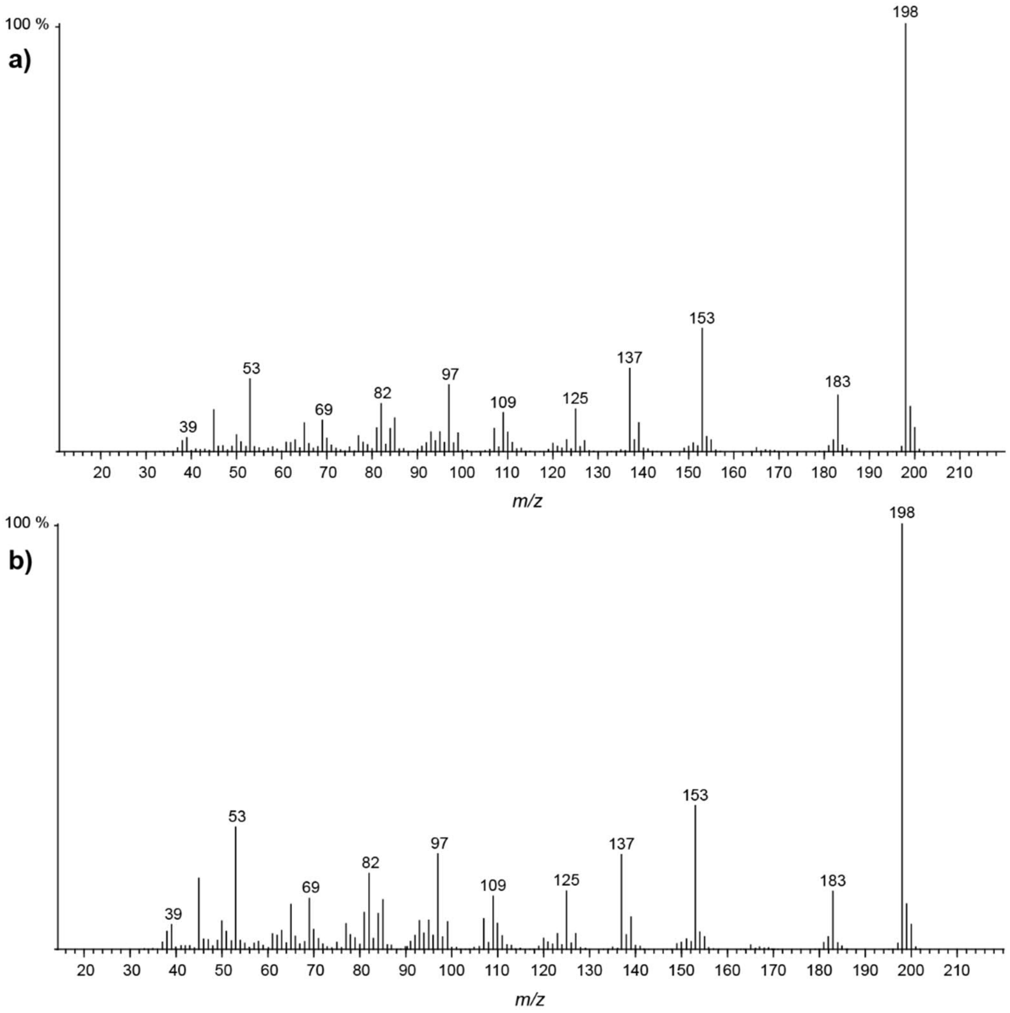

Figure S6. Comparison of the mass spectra of **a)** the natural compound **A** and **b) 9**.

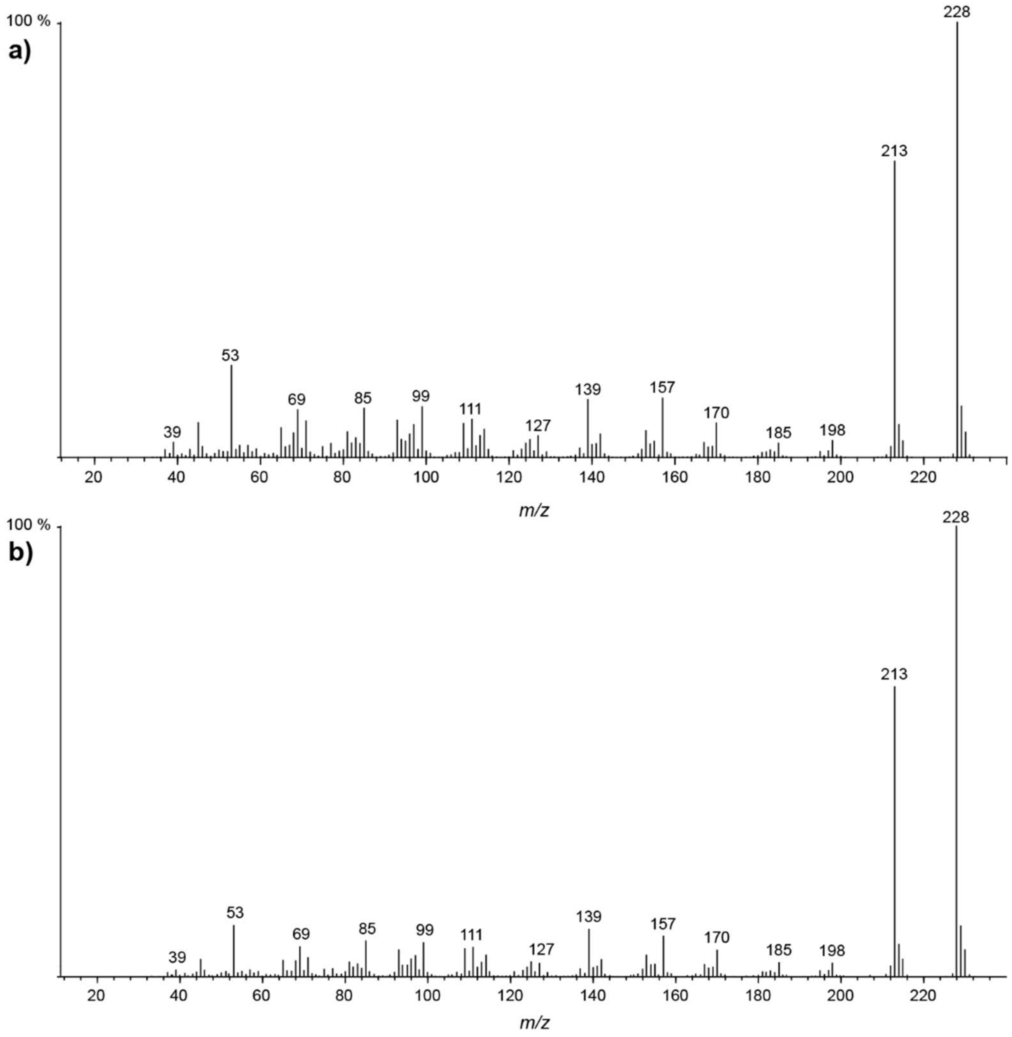

Figure S7. Comparison of the mass spectra of **a)** the natural compound **B** and **b) 10**.

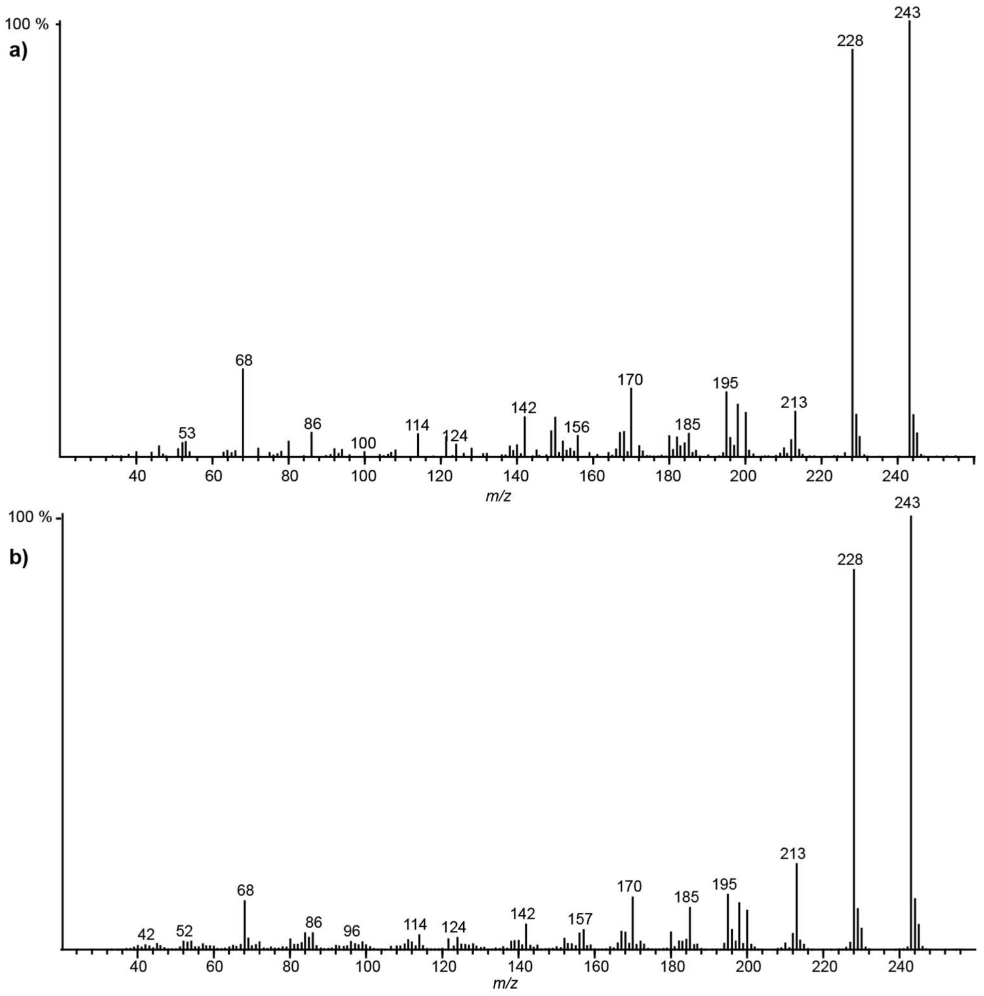

Figure S8. Comparison of the mass spectra of **a)** the natural compound **C** and **b) 11**.

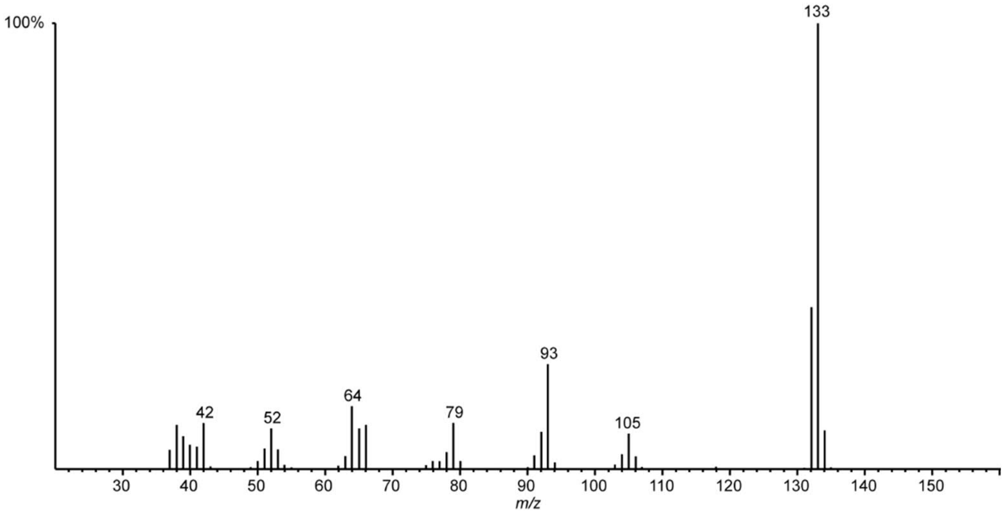

Figure S9. Mass spectra of the natural compound **19**.

Antimicrobial Activity Test

Table S2: Antimicrobial testing of compounds 9–11, 19. Minimum inhibitory concentrations (MICs) were determined in liquid broth according to EUCAST guidelines.

indicator strain	MIC [µg/mL]				
	19	9	10	11	reference antibiotic
Acinetobacter baumannii DSM-30008	>64	>64	>64	>64	0.25[b]
Bacillus subtilis DSM-10	>64	>64	>64	>64	0.25[c]
Citrobacter freundii DSM-30039	>64	>64	>64	>64	≤0.03[b]
Escherichia coli BW25113 (wild type)	>64	>64	>64	>64	0.0125[b]
E. coli JW0451-2 (Δ*acrB*)	>64	>64	>64	>64	≤ 0.003[b]
E. coli K12 Δ*tolC*[a]	>64	>64	>64	>64	0.003[b]
Mycobacterim smegmatis mc^{2}155	>64	>64	>64	>64	32[d]
Staphylococcus aureus Newman[a]	>64	>64	>64	>64	2[c]
Candida albicans DSM-1665[a]	>64	>64	>64	>64	1[e]
Cryptococcus neoformans DSM-11959	>64	>64	>64	>64	4[e]
Mucor hiemalis DSM-2656	>64	>64	>64	>64	n.d.
Wickerhamomyces anomala DSM-6766[a]	>128	128	>128	>128	1[e]

[a]These strains were used to additionally determine the activity of compounds in a disc (6 mm diameter) diffusion assays. Only compounds 19 and 9 showed some inhibitory activity against *W. anomala* with a diameter of inhibition zone of 8 mm and 10 mm, respectively. [b]Ciprofloxacin. [c]Vancomycin. [d]Rifampicin. [e]Amphotericin B.

Material and Methods

General Experimental Procedures.

All reactions were performed in oven-dried glassware under a nitrogen atmosphere. Solvents were dried according to standard procedures. Column chromatography: silica 60 (0.063–0.200 mm, 70–230 mesh ASTM). Thin layer chromatography (TLC): Polygram® SIL G/UV silica 60, 0.20 mm. Compounds were stained with potassium permanganate solution. IR spectra were measured on a Bruker Tensor 27 (diamond-ATR) or Agilent Technologies 7890B gas chromatograph equipped with an HP5 phase (Agilent Technologies, 30 m, 0.25 mm i.d. 0.25 µm film thickness) connected to a Dani Instruments DiscovIR DDFTIRInterface. NMR spectra were recorded either on Avance III HD 300N (^{1}H NMR: 300 MHz, ^{13}C NMR: 76 MHz), AVII 400 (^{1}H NMR: 400 MHz, ^{13}C NMR: 101 MHz), or AVIIIHD-500 MHz (^{1}H NMR: 500 MHz, ^{13}C NMR: 125 MHz) instruments. Mass spectra were recorded with a combination of an Agilent Technologies 5977B gas chromatograph connected to an Agilent Technologies 8860 Series MSD. Gas chromatographic retention indices were calculated against a series of *n*-alkanes according to van den Dool and Kratz[3] using a standard HP-5 phase (Agilent Technologies, 30 m, 0.25 mm i.d. 0.25 µm film thickness). HRMS was performed using an Exactive GC orbitrap mass spectrometer (ThermoScientific, Bremen, Germany). The resolution was set to 60,000 (FWHM; instrument setting at 200 u). The mass range was 50–650 u and 2 micro scans were averaged per data scan. Automated gain control (AGC target) was set to 1×10^{6} and maximum inject time was set to "auto." Auxiliary temperatures were set to 290 °C for both transfer lines 1 and 2 and the temperature of the electron ionization source was set to 220 °C. EI was performed at 70 eV energy in positive mode. Helium (carrier gas) and nitrogen (supply for the C-Trap) were equipped with gas purification cartridges to trap moisture and organic impurities of the gases (Thermo Scientific, Bremen, Germany). Column bleed ion at 207.03235 u was used as lock mass for internal mass calibration of the data. For chemical ionization in positive mode (CIP), methane (99.995%) was used as CI-gas at a flow rate of 1.5 mL/min. Compound **19** was purchased from ABCR (Karlsruhe, Germany).

DFT-calculation

DFT-based calculations of IR signals were performed, as described previously.[4] All molecular mechanical calculations were performed using Spartan '18 (Version 1.4.4). All conformational searches used the MMFF force field method[5] and were done in the gas phase, with a 10 $kJmol^{-1}$ upper energy limit. Typically, the lowest energy conformer was 4 – 5 $kJmol^{-1}$ lower than other conformers, so the analyses could be simplified by discounting the higher energy conformers. Quantum mechanical calculations were carried out using Gaussian09 [6] and employed the

B3LYP functional [7] and 6-31G(d,p) basis set for all calculations. The calculated IR spectra were scaled by a factor of 0.97.

Springtails

Ceratophysella denticulata was cultured for several weeks in our laboratory before extraction. A mixture of plaster of Paris and activated charcoal (10:1) was used as substrate. Baker's yeast was fed *ad libitum*. For extraction, about 50 individuals of all life stages were covered with ultrapure pentane (Merck, Suprasolv). After 30 min., the solvent was removed, giving the pentane extract. The springtails were then covered with ultrapure dichloromethane (Merck, Suprasolv), and the solvent was removed after 30 min, leading to the dichloromethane extract. The remaining springtails were stored under a new batch of dichloromethane. These extracts were stored at –78 °C until analysis. After three months, the solvent was removed again and the springtails were covered with methanol (Merck). The origin and sample preparation of *Hypogastrura viatica* has been described earlier.[8]

Bioassay

Fifteen workers of *Lasius niger* were placed in a petri dish (15 cm diameter), deprived of food for 7 days, but provided with tapwater. After starvation of 7 days, the bioassay was started by offering 7 µL of each test solution and control solution simultaneously. Both solutions consisted of 40 % honey dissolved in condensed milk. For the test solution, 0.1 % w/w of the compound to be tested was added. Since the concentration of the compounds in *C. denticulata* was unknown we used the concentration of sigillin, a known deterrent from *Ceratophysella sigillata*, which occurs in a concentration of 0.2%w/w. To be conservative we used 0.1%w/w in our assay.[9] The concentration of the test compounds in 1 µl test solution was about 6 nMol. The number of ants feeding upon test and control solution was recorded every 20 seconds for 5 minutes. This bioassay was repeated 10 times for 4-methoxy-5-methylthiobenzo-1,3-dioxolane (**9**) and 5,6,7-trimethoxybenzo-1,3-oxathiolane (**10**), 12 times for 4-amino-5,6,7-trimethoxybenzo-1,3-oxathiolane (**11**), and 13 times for 2-methylimidazo[4,5-c]pyridine (**19**).

Synthetic procedures

*5-Bromo-4-hydroxybenzo-1,3-dioxolane (**13**)*

Bromine (185 µL, 3.620 mmol, 1.0 eq.) was added to a stirred solution of **12** (500 mg, 3.620 mmol, 1.0 eq.) and silver trifluoroacetate (800 mg, 3.620 mmol, 1.0 eq.) in $CHCl_3$ (25 mL) at 0 °C. The mixture was stirred for 1 h at 10 – 15 °C.[10] Afterward, the reaction was quenched by

the addition of sat. Na_2SO_3 solution (25 mL). The phases were separated and the aqueous layer was extracted with $CHCl_3$ (3x 25 mL). The combined organic phases were dried over $MgSO_4$ and concentrated under reduced pressure. The residue was purified by flash chromatography (CH_2Cl_2/MeOH; 40:1). **13** was obtained as a colorless solid (724 mg, 3.336 mmol, 92%). FT-IR: ν / cm^{-1} = 3298, 2904, 1629, 1464, 1361, 1309, 1251, 1220, 1156, 1101, 1059, 1012, 926, 864, 790, 696, 599, 582, 561, 541. ^{1}H-NMR: (300 MHz, $CDCl_3$) δ / ppm = 6.95 (d, J = 8.4 Hz, 1H), 6.41 (d, J = 8.4 Hz, 1H), 6.01 (s, 2H), 5.32 (s, 1H). ^{13}C-NMR, DEPT: (76 MHz, $CDCl_3$) δ 148.9 (C_{Ar}), 137.1 (C_{Ar}), 134.8 (C_{Ar}), 124.3 (CH_{Ar}), 103.5 (C_{Ar}), 102.8 (CH_{Ar}), 102.3 (CH_2). EI-MS (70 eV): m/z = 218 (90, $[M(^{81}Br)]^+$), 217 (79), 216 (100, $[M(^{79}Br)]^+$), 215 (66), 106 (57), 81 (52), 79 (78), 53 (79), 51 (60), 50 (75). HR-MS: m/z = 214.93393 $[M(^{79}Br)–H]^+$ (calc.: 214.93383).

*5-Bromo-4-methoxybenzo-1,3-dioxolane (**14**)*

NaH (60% dispersion in mineral oil, 145 mg, 3.619 mmol, 1.1 eq.) was added to a stirred solution of **13** (714 mg, 3.290 mmol, 1.0 eq.) in DMSO (10 mL).[11] The mixture was stirred for 30 min at room temperature (rt). Afterward, MeI (246 µL, 3.948 mmol) was added to the mixture and the mixture was stirred further for 1 h at rt. The reaction was quenched by the addition of water (20 mL) and extracted with Et_2O (3x 20 mL). The combined organic phases were washed with brine (20 mL), dried over $MgSO_4$, and concentrated under reduced pressure. The residue was purified by flash chromatography (Pe/Et_2O; 40:1). Compound **14** was obtained as a colorless oil that crystallized over time (741 mg, 3.207 mmol, 97%). FT-IR: ν / cm^{-1} = 2943, 2892, 2777, 1622, 1599, 1498, 1472, 1452, 1428, 1335, 1262, 1232, 1126, 1101, 1069, 1033, 975, 934, 868, 789, 690, 656, 605, 549. ^{1}H-NMR: (300 MHz, $CDCl_3$) δ / ppm = 6.99 (d, J = 8.3 Hz, 1H), 6.44 (d, J = 8.3 Hz, 1H), 5.96 (s, 2H), 4.03 (s, 3H). ^{13}C-NMR, DEPT: (75 MHz, $CDCl_3$) δ / ppm = 149.1 (C_{Ar}), 140.9 (C_{Ar}), 137.8 (C_{Ar}), 125.5 (CH_{Ar}), 107.1 (C_{Ar}), 104.1 (CH_{Ar}), 101.7 (CH_2), 60.3 (CH_3). EI-MS (70 eV): m/z = 232 (97, $[M(^{81}Br)]^+$), 231 (32), 230 (100, $[M(^{79}Br)]^+$), 187 (29), 159 (34), 157 (31), 77 (28), 65 (51), 53 (88), 50 (63). HR-MS: m/z = 229.95731 $[M(^{79}Br)]^+$ (calc.: 229.95731).

*4-Methoxy-5-methylthiobenzo-1,3-dioxolane (**9**)*

A stirred solution of **14** (630 mg, 2.727 mmol, 1.0 eq.) in THF (1 mL) was degassed using the freeze-pump-thaw method. *n*BuLi (1.6 M in hexane, 2.560 mL, 4.090 mmol, 1.5 eq.) was added to the stirred solution at –100 °C.[10] The resulting solution was stirred for 30 min at –100 °C. Afterward, MeSSMe (412 µL, 4.635 mmol, 1.7 eq.) was added to the solution. The resulting mixture was stirred for 1 h while it was allowed slowly to warm up in the cooling bath. The reaction was quenched by the addition of sat. NH_4Cl solution (20 mL) and extracted with Et_2O (3x 20 mL). The combined organic phases were washed with brine (20 mL), dried over $MgSO_4$,

and concentrated under reduced pressure. The residue was purified by flash chromatography (pentane/CH_2Cl_2; 10:1). **9** was obtained as a colorless oil that crystallized over time (418 mg, 2.109 mmol, 77%). FT-IR: ν / cm^{-1} = 3006, 2988, 2904, 2891, 1618, 1497, 1471, 1462, 1428, 1338, 1257, 1235, 1205, 1114, 1068, 1033, 977, 936, 889, 788. ^{1}H-NMR: (300 MHz, $CDCl_3$) δ / ppm = 6.74 (d, J = 8.2 Hz, 1H), 6.52 (d, J = 8.1 Hz, 1H), 5.93 (s, J = 7.2 Hz, 2H), 4.05 (s, 3H), 2.38 (s, 3H). ^{13}C-NMR, DEPT: (75 MHz, $CDCl_3$) δ / ppm = 148.5 (C_{Ar}), 141.9 (C_{Ar}), 137.0 (C_{Ar}), 122.5 (C_{Ar}), 122.2 (CH_{Ar}), 103.2 (CH_{Ar}), 101.3 (CH_2), 60.0 (OCH_3), 17.0 (SCH_3). EI-MS (70 eV): *m/z* = 198 (100, $[M]^+$), 153 (40), 137 (29), 97 (35), 85 (19), 82 (28), 69 (20), 65 (19), 53 (53), 45 (32). HR-MS: *m/z* = 198.03458 $[M]^+$ (calc.: 198.03452). *I* = 1602 (HP5 phase).

*2,3,4-Trimethoxyphenol (**16**)*

H_2SO_4 (conc., 90 µL) was added to a stirred solution of **15** (1.000 g, 5.097 mmol, 1.0 eq.) and H_2O_2 (30 %w/V, 563 µL) in MeOH (6 mL) at 0 °C.[12] The resulting mixture was stirred for 1 h at rt. Afterward, the reaction was quenched by the addition of sat. $NaHCO_3$ solution (5 mL). The mixture was extracted with CH_2Cl_2 (3x 5 mL). The combined organic phases were washed with brine (5 mL), dried over $MgSO_4$, and concentrated under reduced pressure. The residue was purified by flash chromatography (CH_2Cl_2/MeOH; 40:1). Phenol **16** was obtained as a yellow oil (897 mg, 4.870 mmol, 96%). FT-IR: ν / cm^{-1} = 3422, 2940, 2834, 1599, 1490, 1477, 1426, 1358, 1265, 1195, 1157, 1086, 1045, 1007, 955, 901, 796, 734, 679, 657, 622, 5583, 571, 529. ^{1}H-NMR: (300 MHz, $CDCl_3$) δ / ppm = 6.63 (d, J = 9.0 Hz, 1H), 6.55 (d, J = 9.0 Hz, 1H), 5.42 (s, 1H), 3.96 (s, 3H), 3.89 (s, J = 1.2 Hz, 3H), 3.81 (s, 3H). ^{13}C-NMR, DEPT: (76 MHz, $CDCl_3$) δ / ppm = 147.1 (C_{Ar}), 143.4 (C_{Ar}), 142.4 (C_{Ar}), 140.6 (C_{Ar}), 108.6 (CH_{Ar}), 107.7 (CH_{Ar}), 61.4 (CH_3), 61.0 (CH_3), 56.7 (CH_3). EI-MS (70 eV): *m/z* = 184 (100, $[M]^+$), 169 (75), 141 (14), 126 (56), 123 (30), 109 (13), 95 (15), 83 (15), 69 (15), 55 (24). HR-MS: *m/z* = 184.07272 $[M]^+$ (calc.: 184.07301).

*6-Mercapto-2,3,4-trimethoxyphenol (**17**)*

H_2SO_2 (conc., 518 µL) was added to a stirred solution of **16** (1.491 g, 8.095 mmol, 1.0 eq.), DMSO (690 µL, 9.714 mmol, 1.2 eq.) and KSCN (0.944 g, 9.714 mmol, 1.2 eq.) in dimethylformamide (DMF, 15 mL).[13] The mixture was stirred for 2 h at 80 °C. An aqueous solution of LiCl (10 %, 15 mL) was added. The mixture was extracted with CH_2Cl_2 (3x 15 mL). The combined organic phases were dried over $MgSO_4$ and concentrated under reduced pressure. The residue was taken up in THF (50 mL). An aqueous solution of LiOH (1 M, 15 mL) was added and the resulting mixture was stirred for 2 h at rt.[14] The reaction was quenched with water (50 mL) and extracted with CH_2Cl_2 (3x 50 mL). The combined organic phases were washed with brine (50 mL), dried over $MgSO_4$, and concentrated under reduced pressure. An NMR spectrum of the crude residue indicated that it was not the desired **17** but rather the

thiocarbamate formed by the addition of water to thiocyanate. To this residue dissolved in THF (10 mL) was added $LiAlH_4$ (290 mg, 7.641 mmol). The mixture was stirred for 0.5 h at rt and sat. Rochelle solution (10 mL) was added. After stirring an additional 0.5 h, the mixture was extracted with Et_2O (3x 10 mL). The combined organic phases were dried over $MgSO_4$ and concentrated under reduced pressure. The residue was purified by flash chromatography (CH_2Cl_2/MeOH; 40:1). Phenol **17** was obtained as a yellow solid (746 mg, 3.450 mmol, 43%). FT-IR: ν / cm^{-1} = 3410, 2940, 2834, 1594, 1490, 1462, 1432, 1415, 1357, 1283, 1189, 1113, 1060, 1020, 969, 908, 825, 673, 572, 550. ^{1}H-NMR: (400 MHz, $CDCl_3$) δ / ppm = 6.69 (s, 1H), 5.90 (s, 1H), 3.93 (s, 3H), 3.91 (s, 3H), 3.73 (s, 3H). ^{13}C-NMR, DEPT: (101 MHz, $CDCl_3$) δ / ppm = 146.7, 144.7, 141.0, 114.6, 112.4, 61.4, 61.3, 56.5. EI-MS (70 eV): m/z = 216 (100, $[M]^+$), 201 (83), 173 (14), 158 (48), 155 (15), 129 (15), 101 (16), 85 (20), 69 (14), 53 (14). 216.04507 $[M]^+$ (calc.: 216.04508).

*5,6,7-Trimethoxybenzo-1,3-oxathiolane (**10**)*

Phenol **17** (2.400 g, 11.098 mmol, 1.0 eq.) in DMF (20 mL) was added to a stirred solution of Cs_2CO_3 (10.848 g, 33.295mmol, 3.0 eq.) and CH_2I_2 (1.341 mL, 4.460 mmol, 1.5 eq.) in DMF (120 mL) at 80 °C.[15] The reaction mixture was stirred for 2 h at 80 °C and 8 h at rt. The white precipitate was filtered off and the filtrate was extracted with CH_2Cl_2 (3x 100 mL). The combined organic phases were washed with brine (100 mL), dried over $MgSO_4$, and concentrated under reduced pressure. The residue was purified by flash chromatography (pentane/Et_2O; 5:1). Oxathiolane **10** was obtained as a yellow solid (1.309 g, 5.735 mmol, 52%). FT-IR: ν / cm^{-1} = 3426, 3343, 2991, 2937, 1627, 1483, 1463, 1416, 1389, 1307, 1293, 1254, 1197, 1142, 1084, 1030, 1012, 949, 902, 831, 759, 715. ^{1}H-NMR: (300 MHz, $CDCl_3$) δ / ppm = 5.71 (s, J = 1.9 Hz, 2H), 3.89 (s, J = 3.4 Hz, 3H), 3.84 (s, J = 1.3 Hz, 3H), 3.81 (s, J = 1.1 Hz, 3H), 3.57 (s, J = 7.1 Hz, 2H). ^{13}C-NMR, DEPT: (76 MHz, $CDCl_3$) δ / ppm = 145.1 (C_{Ar}), 144.9 (C_{Ar}), 136.3 (C_{Ar}), 130.4 (C_{Ar}), 105.7 (C_{Ar}), 61.6 (CH_3), 61.5 (CH_3), 60.9 (CH_3). EI-MS (70 eV): m/z = 244 (12), 243 (100, $[M]^+$), 229 (9), 228 (88), 213 (20), 198 (11), 195 (13), 185 (10), 170 (12), 68 (11). HR-MS: m/z = 228.04496 $[M]^+$ (calc.: 228.04508). I= 1803 (on an HP5 phase).

*5,6,7-Trimethoxy-4-nitrobenzo-1,3-oxathiolane (**18**)*

HNO_3 (65%, 113 µL) was added to a stirred solution of **10** (64 mg, 0.280 mmol, 1.0 eq.) in CH_2Cl_2 (3 mL).[16] The resulting mixture was stirred for 1.5 h at rt. The reaction was quenched by the addition of sat. $NaHCO_3$ solution (5 mL). The phases were separated and the aqueous phase was extracted with CH_2Cl_2 (3x 5 mL). The combined organic phases were dried over $MgSO_4$ and concentrated under reduced pressure. GC/MS analysis of the crude mixture

showed a mixture of **18** and the corresponding sulfoxide. The crude product was therefore dissolved in MeCN (1 mL) and treated with Tf_2O (16 µL, 0.096 mmol, 1.1 eq.) and KI (36 mg, 0.219 mmol, 2.5 eq.) at –40 °C. After stirring the mixture for 20 min at –40 °C, the reaction was quenched by the addition of sat. $Na_2S_2O_4$ solution (5 mL). The mixture was extracted with CH_2Cl_2 (3x 5 mL). The combined organic phases were dried over $MgSO_4$ and concentrated under reduced pressure. The residue was purified by flash chromatography (pentane/Et_2O; 1:1). Oxathiolane **18** was obtained as a yellow solid (25 mg, 0.092 mmol, 33%). FT-IR: ν / cm^{-1} = 2941, 1660 ,1570, 1507, 1467, 1404, 1329, 1300, 1271, 1194, 1116, 1067, 1012, 971, 938, 896, 849, 813, 762, 678, 626, 554, 538. ^{1}H-NMR: (500 MHz, $CDCl_3$) δ 5.70 (s, 2H), 4.05 (s, 3H), 3.95 (s, 3H), 3.89 (s, 3H). ^{13}C-NMR, DEPT: (126 MHz, $CDCl_3$) δ 145.2, 145.1, 144.0, 143.4, 123.2, 76.3, 62.6, 61.9, 61.2. EI-MS (70 eV): *m/z* = 273 (100, $[M]^+$), 197 (16), 152 (28), 99 (26), 96 (15), 80 (16), 70 (15), 69 (17), 59 (17), 45 (15). HR-MS: *m/z* = 273.03006 $[M]^+$ (calc.: 273.03016).

*4-Amino-5,6,7-trimethoxybenzo-1,3-oxathiolane (**11**)*

$SnCl_2$ (124 mg, 0.549 mmol, 6.0 eq.) was added to a stirred solution of **18** (25 mg, 0.091 mmol, 1.0 eq.) in EtOH (2 mL).[17] The resulting mixture was stirred for 3 h at 60 °C. After complete conversion, the reaction was quenched by the addition of sat. $NaHCO_3$ solution (10 mL). The resulting mixture was extracted with CH_2Cl_2 (3x 10 mL). The combined organic phases were dried over $MgSO_4$ and concentrated under reduced pressure. The residue was purified by flash chromatography (CH_2Cl_2/NEt_3; 200:1). Amine **11** was obtained as a red oil (17mg, 0.070 mmol, 77%). FT-IR: ν/ cm^{-1} = 3426, 3343, 2991, 2937, 1627, 1483, 1463, 1416, 1389, 1307, 1293, 1254, 1197, 1142, 1084, 1030, 1012, 949, 902, 831, 759, 715. ^{1}H-NMR: (300 MHz, $CDCl_3$) *δ* / ppm = 5.71 (s, *J* = 1.9 Hz, 2H), 3.89 (s, *J* = 3.4 Hz, 3H), 3.84 (s, *J* = 1.3 Hz, 3H), 3.81 (s, *J* = 1.1 Hz, 3H), 3.57 (s, *J* = 7.1 Hz, 2H). ^{13}C-NMR, DEPT: (76 MHz, $CDCl_3$) *δ* / ppm = 145.1 (C_{Ar}), 144.9 (C_{Ar}), 136.3 (C_{Ar}), 130.4 (C_{Ar}), 105.7 (C_{Ar}), 61.6 (CH_3), 61.5 (CH_3), 60.9 (CH_3). EI-MS (70 eV): m/z = 244 (12), 243 (100, $[M]^+$), 229 (9), 228 (88), 213 (20), 198 (11), 195 (13), 185 (10), 170 (12), 68 (11). HR-MS: *m/z* = 243.05605 $[M]^+$ (calc.: 243.05598). *I* = 1962 (HP5 phase).

*Benzo-1,3-dioxolane-5,6-diol (**S26**)*

Sesamol (**S25**, 1 g, 7.240 mmol, 1.0 eq.) in MeOH (25 mL) was added to a stirred solution of freshly prepared Fremy's salt (4.857 g, 18.100 mmol, 2.5 eq.) and KH_2PO_4 (3.251 g, 23.892 mmol, 3.3 eq.) in water (270 mL) at 0 °C. The reaction mixture was stirred for 30 min at 0 °C. Afterward, it was extracted with EtOAc (4x40 mL). A solution of $Na_2S_2O_4$ (5.294 g, 30.408 mmol, 4.2 eq.) in water (20 mL) was added to the combined organic phases, acidified with HCl (1 M) and extracted with EtOAc (3x30 mL). The combined organic phases were washed with brine (30 mL), dried over $MgSO_4$, and concentrated under reduced pressure. The diol **S26** was

obtained as a violet solid (848 mg, 5.506 mmol, 76%), which was used directly without further purification. FT-IR: ν/ cm^{-1} =3436, 3343, 3066, 2892, 1656, 1501, 1460, 1403, 1362, 1323, 1210, 1150, 1031, 930, 854, 776, 759, 712, 642, 586, 564, 544^{1}H-NMR: (300 MHz, acetone) δ / ppm = 67.50 (s, 1H), 6.48 (s, 1H), 5.89 – 5.76 (m, 1H). ^{13}C-NMR, DEPT: (76 MHz, acetone) δ / ppm = 140.9 (C_q), 139.6 (C_q), 101.3 (CH), 98.8 (CH_2). EI-MS (70 eV): m/z = 154 (100, [M]$^+$), 153 (62), 53 (52), 69 (36), 39 (26), 40 (24), 96 (23), 50 (19), 77 (14), 42 (13). HR-MS: *m/z* = 154.02623 [M]$^+$ (calc.: 154.02606).

*5,6-Dimethoxybenzo-1,3-dioxolane (**S27**)*

S26 (400 mg, 2.595 mmol, 1.0 eq.) was added to a solution of K_2CO_3 (897 mg, 6.488 mmol, 2.5 eq.) in acetone (20 mL) at rt. After stirring for 15 min MeI (339 µL, 5.450 mmol, 2.1 eq.) was added to the solution. The resulting mixture was heated to reflux for 16 h. After cooling to rt, the mixture was filtered over a fritted glass funnel and concentrated under reduced pressure. The residue was taken up in a EtOAc and was filtered again over a plug of Celite. The dioxolane **S27** was obtained as a violet solid (848 mg, 5.506 mmol, 76%).%). FT-IR: ν/ cm^{-1} = 3007, 2937, 1503, 1451, 1435, 1356, 1307, 1210, 1179, 1161, 1090, 1025, 985, 922, 860, 810, 753, 705, 606, 571, 540. ^{1}H-NMR: (300 MHz, $CDCl_3$) δ / ppm = 6.59 (s, 1H), 5.88 (s, *J* = 1.2 Hz, 1H). ^{13}C-NMR, DEPT: (76 MHz, $CDCl_3$) δ / ppm = 143.9 (C_q), 140.8 (C_q), 101.2 (CH_2), 96.3 (CH), 57.2 (CH_3).. EI-MS (70 eV): m/z = 182 (100, [*M*]$^+$), 167 (73), 109 (99), 81 (72), 69 (66), 66 (39), 65 (29), 59 (42), 53 (84), 39 (28). HR-MS: *m/z* = 182.05724 [M]$^+$ (calc.: 182.05736).

*5,6-Dimethoxy-4-(methylthio)benzo-1,3-dioxolane (**S12**)*

A solution of **S27** (50 mg, 0.274 mmol, 1.0 eq.) in THF (1 mL) was degassed by the freeze-pump-thaw technique. *n*BuLi (1.6 M in hexane, 190 µL, 0.302 mmol, 1.1 eq.) and tetramethylethylenediamine (58 µL, 0.384 mmol, 1.4 eq.) were added to the degassed solution at –0 °C. After stirring for 2.5 h at rt, DMDS (38 µL, 0.427 mmol, 1.3 eq.) was added at –78 °C. The reaction mixture was allowed to warm to rt and was quenched by the addition of sat. aqueous NH_4Cl solution (5 mL) after an additional 2 h. The resulting mixture was extracted by Et_2O (3x5 mL). The organic phases were combined, washed with brine (5 mL), dried with MgSO4, and concentrated under reduced pressure. The residue was purified by flash chromatography (pentane/Et_2O; 10:1). The methyl sulfide **S12** was obtained as a colorless liquid (30 mg, 0.131 mmol, 48%). FT-IR: ν/ cm^{-1} = 2962, 2932, 1623, 1469, 1450, 1408, 1365, 1276, 1198, 11174, 1097, 1051, 1031, 988, 942, 912, 846, 815, 767. ^{1}H-NMR: (300 MHz, $CDCl_3$) δ / ppm = 6.49 (s, 1H), 5.94 (s, 2H), 3.81 (s, 3H), 3.81 (s, 3H), 2.50 (s, 3H). ^{13}C-NMR, DEPT: (76 MHz, $CDCl_3$) δ / ppm = 147.8 (C_{Ar}), 143.1 (C_{Ar}), 143.0 (C_{Ar}), 141.4 (C_{Ar}), 113.6 (C_{Ar}),

101.3 (CH_2), 95.3 (CH_{Ar}), 61.2 (CH_3), 57.1 (CH_3), 17.1 (CH_3). EI-MS (70 eV): m/z = 228 (100, [*M*]$^+$), 213 (73), 167 (35), 155 (16), 151 (11), 139 (15), 121 (13), 99 (18), 69 (29), 53 (19). HR-MS: *m/z* = 228.04477 [M]$^+$ (calc.: 228.04508).

NMR-Data

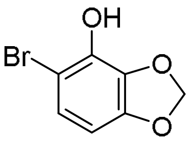

13

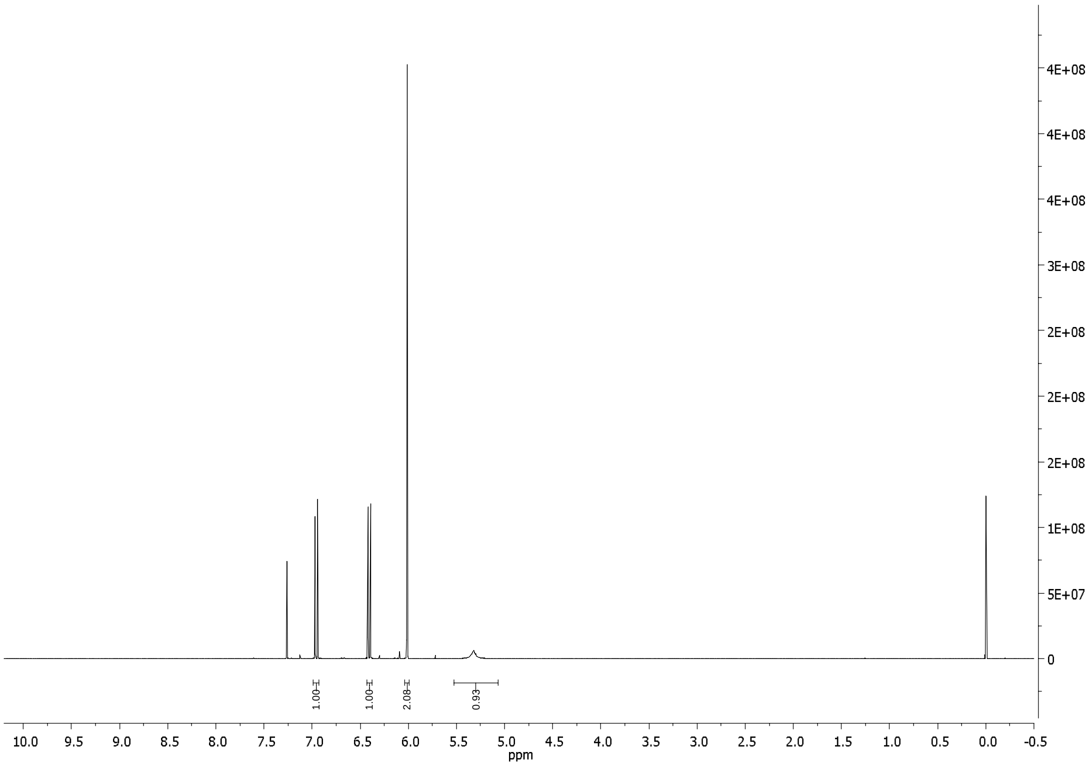

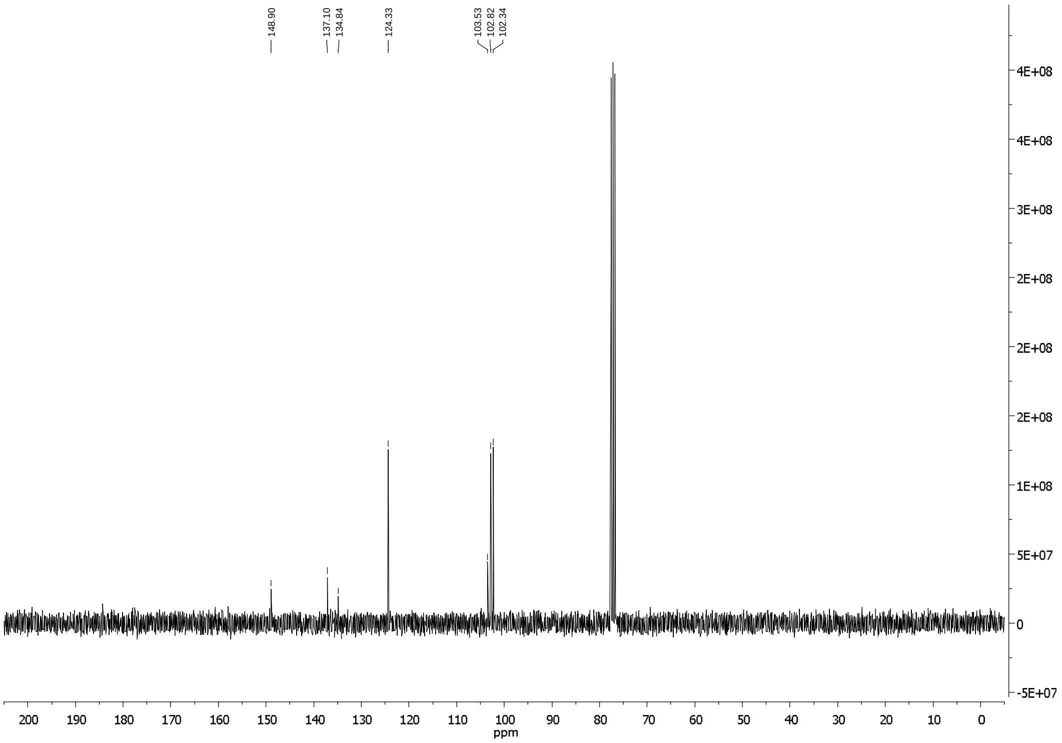

Figure S10. ^{1}H-NMR (300 MHz, $CDCl_3$) and ^{13}C-NMR (76 MHz, $CDCl_3$) of **13**.

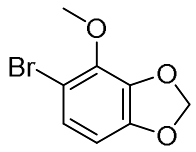

14

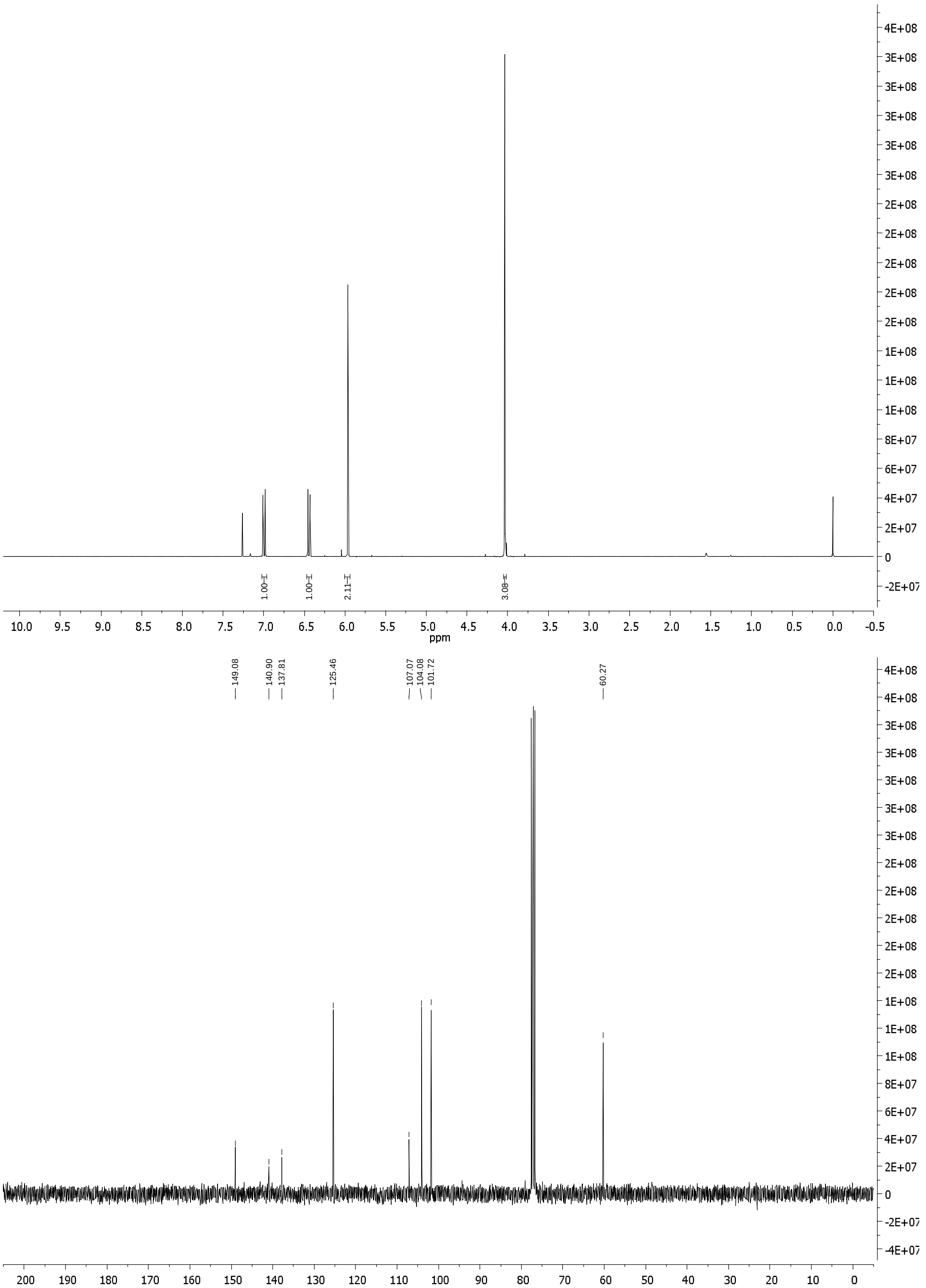

Figure S11. ^{1}H-NMR (300 MHz, $CDCl_3$) and ^{13}C-NMR (76 MHz, $CDCl_3$) of **14.**

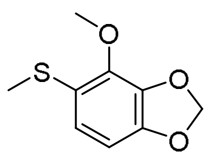

9

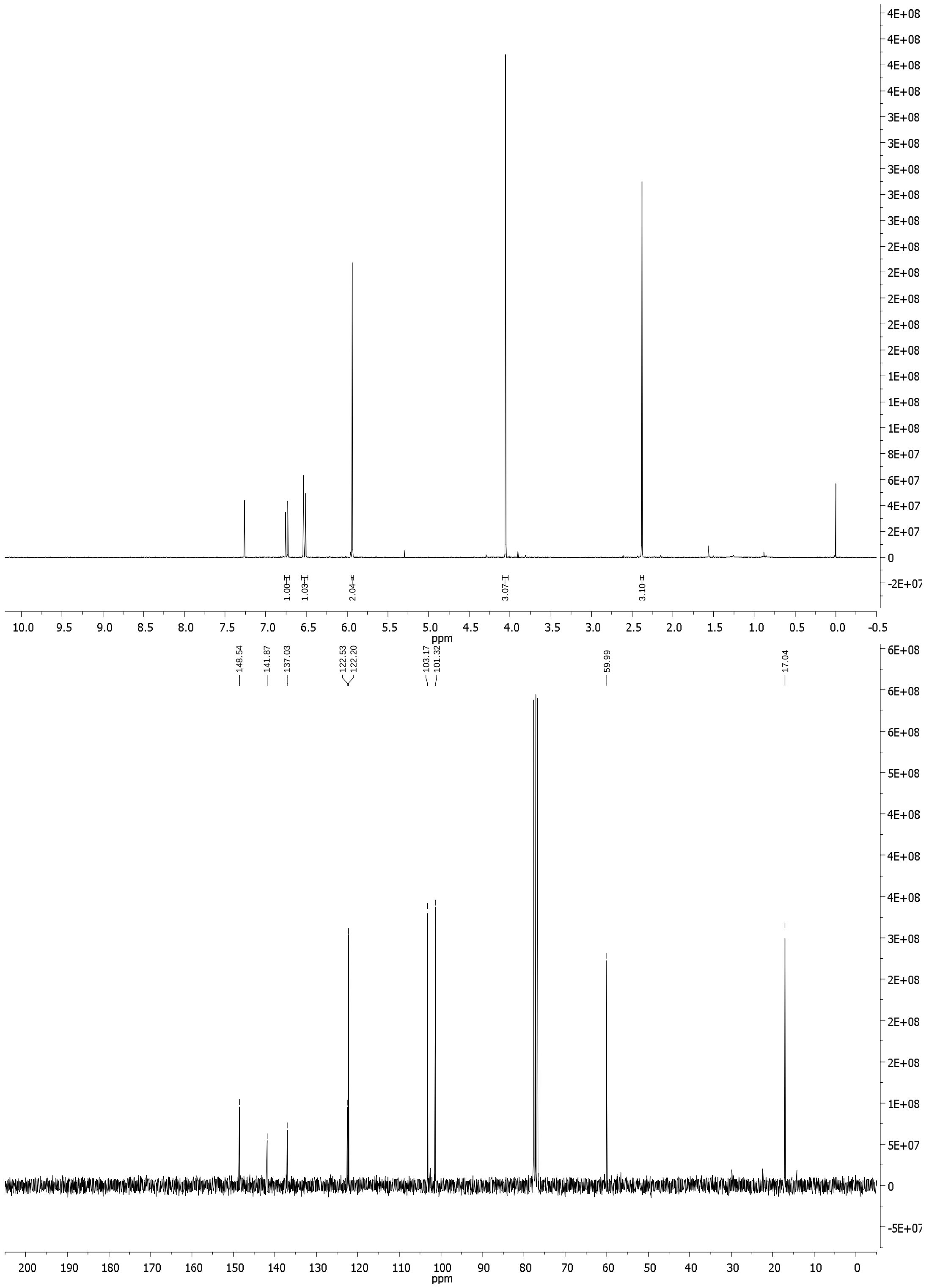

Figure S12. ^{1}H-NMR (300 MHz, $CDCl_3$) and ^{13}C-NMR (76 MHz, $CDCl_3$) of **9**.

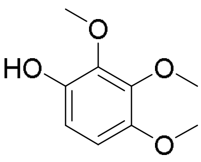

16

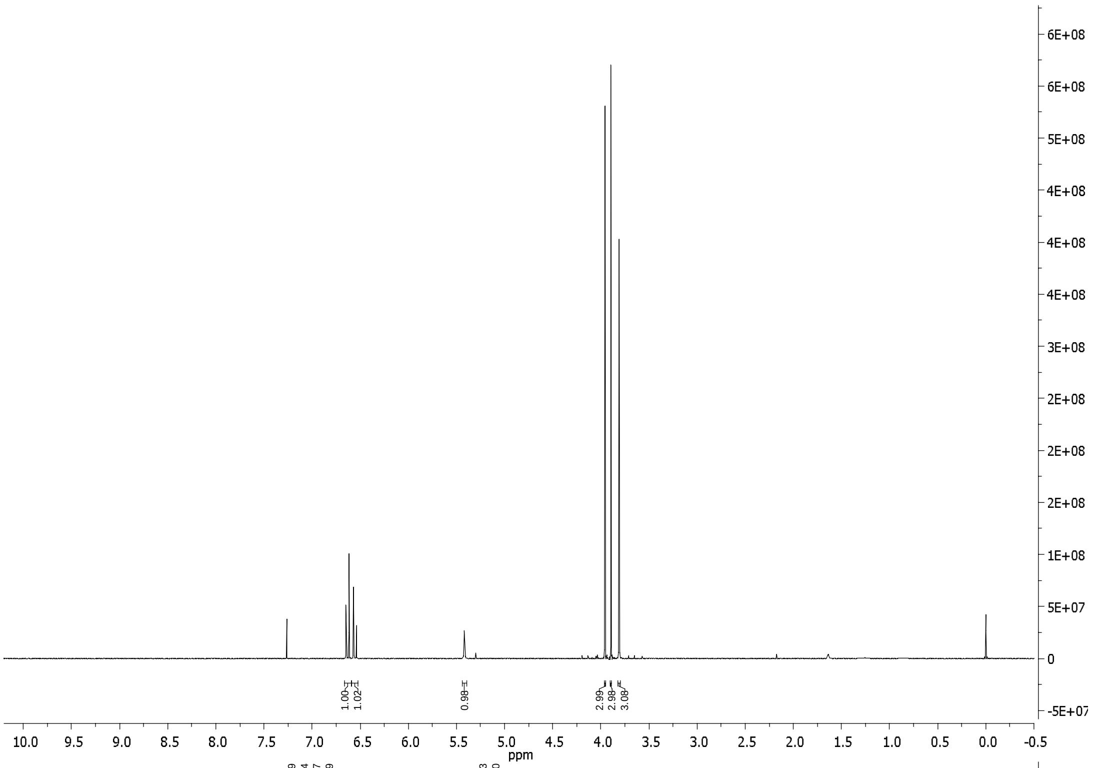

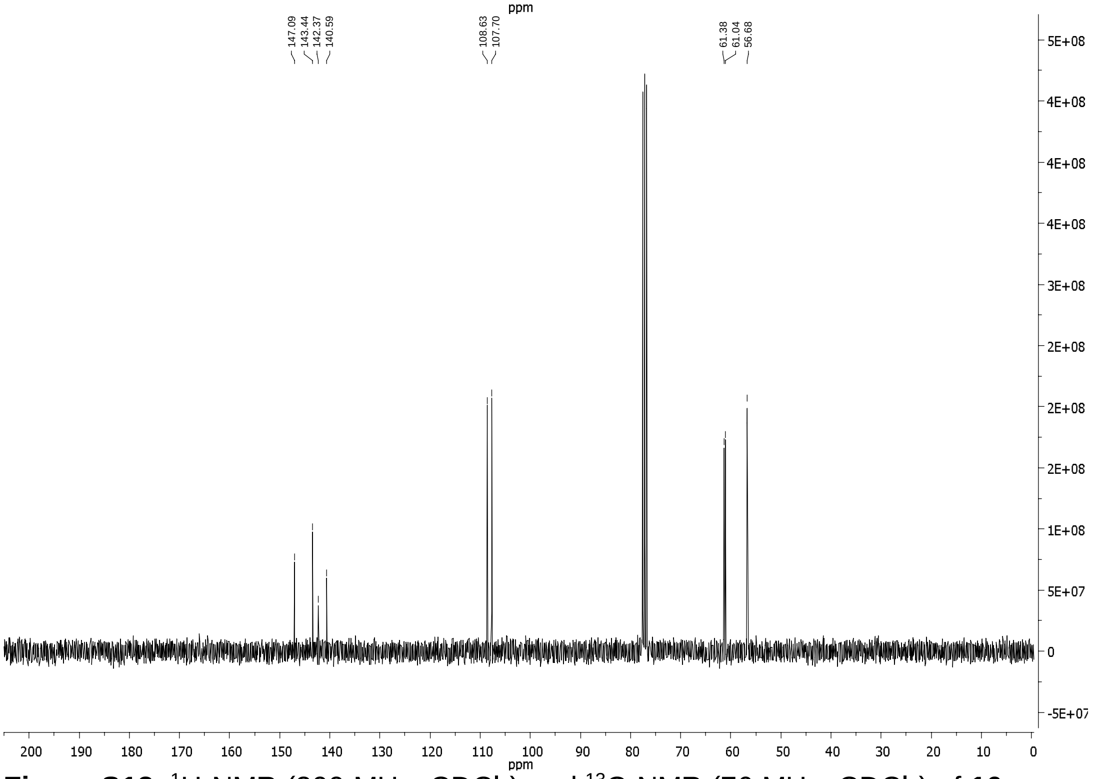

Figure S13. ^{1}H-NMR (300 MHz, $CDCl_3$) and ^{13}C-NMR (76 MHz, $CDCl_3$) of **16**.

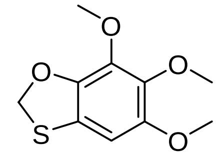

10

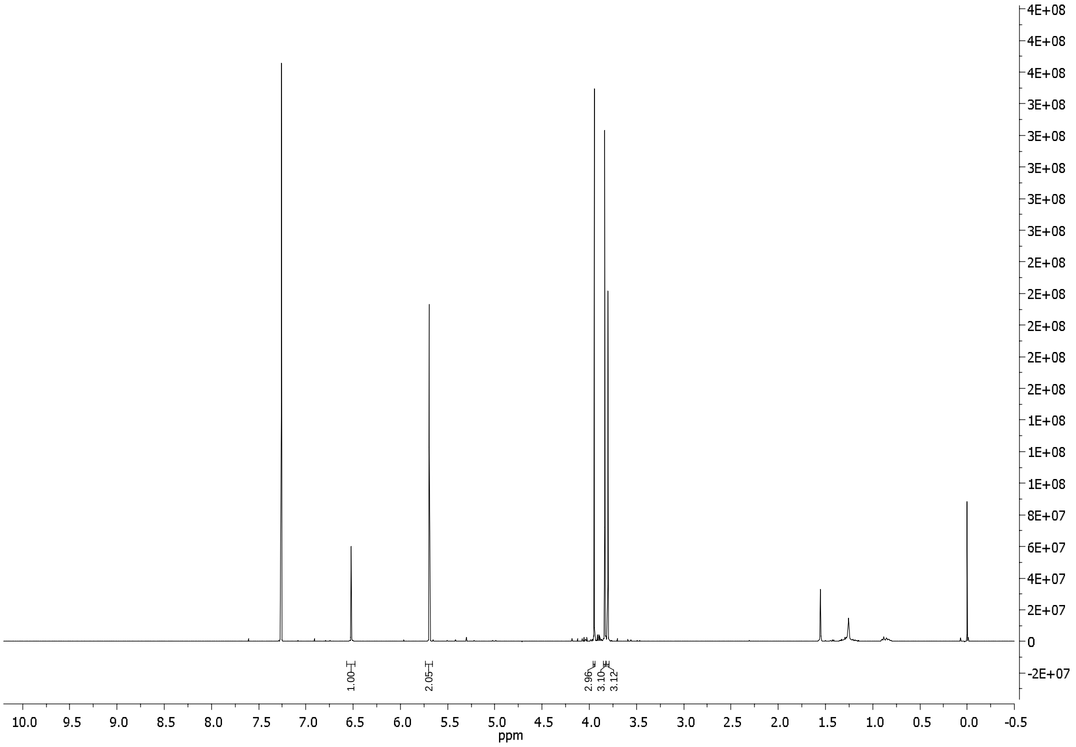

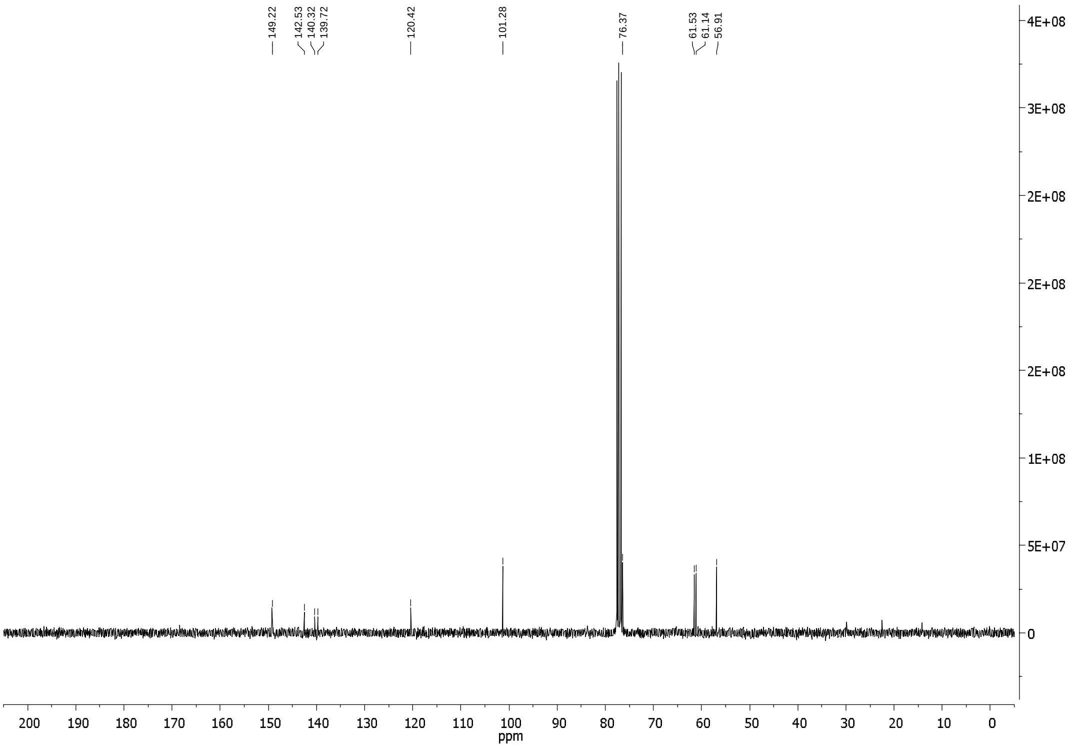

Figure S14. ^{1}H-NMR (300 MHz, $CDCl_3$) and ^{13}C-NMR (76 MHz, $CDCl_3$) of **10**.

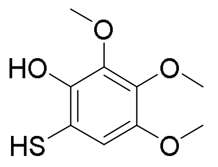

17

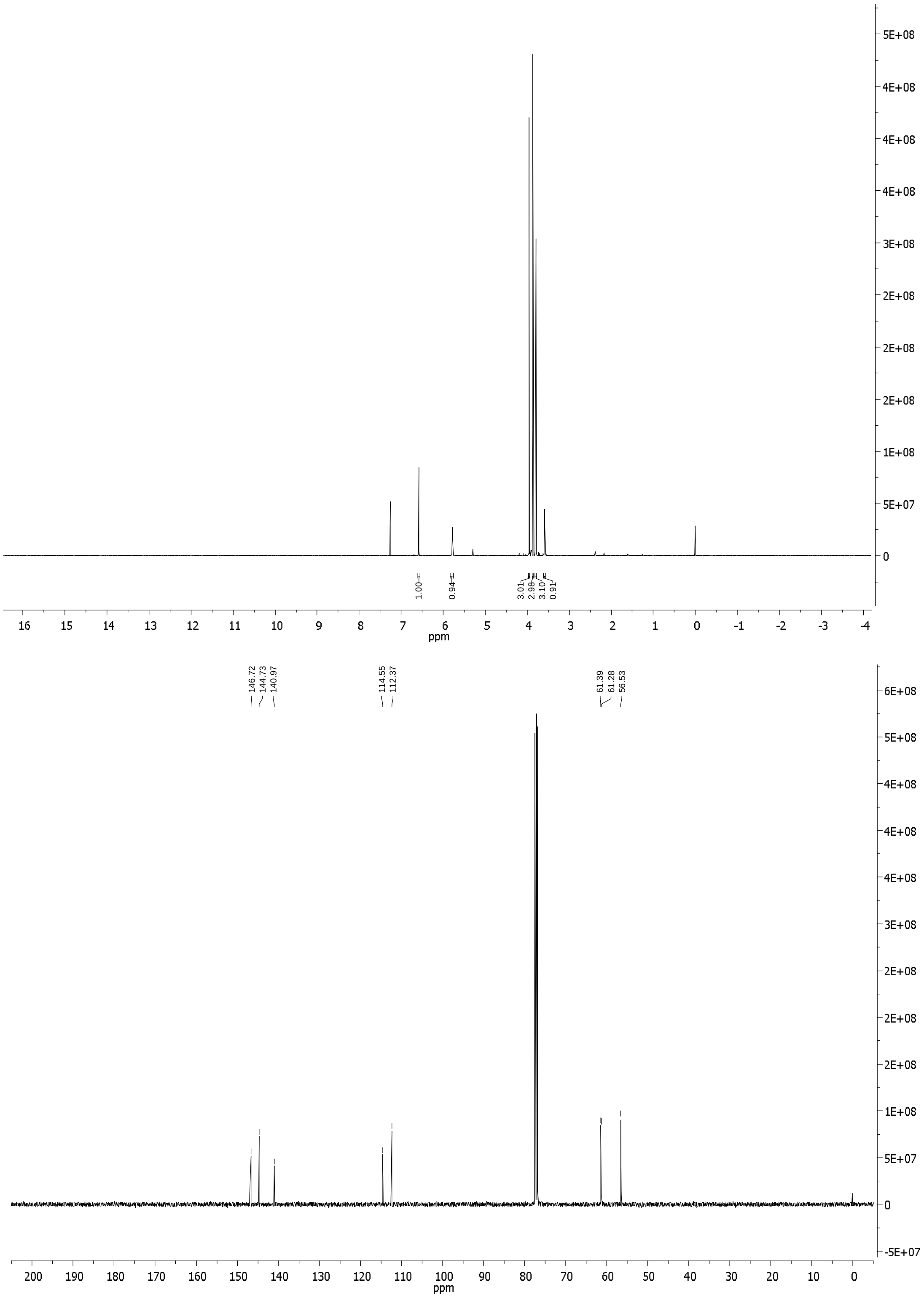

Figure S15. ^{1}H-NMR (300 MHz, $CDCl_3$) and ^{13}C-NMR (76 MHz, $CDCl_3$) of **17**.

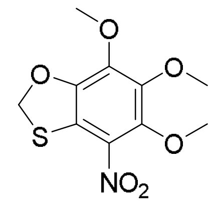

18

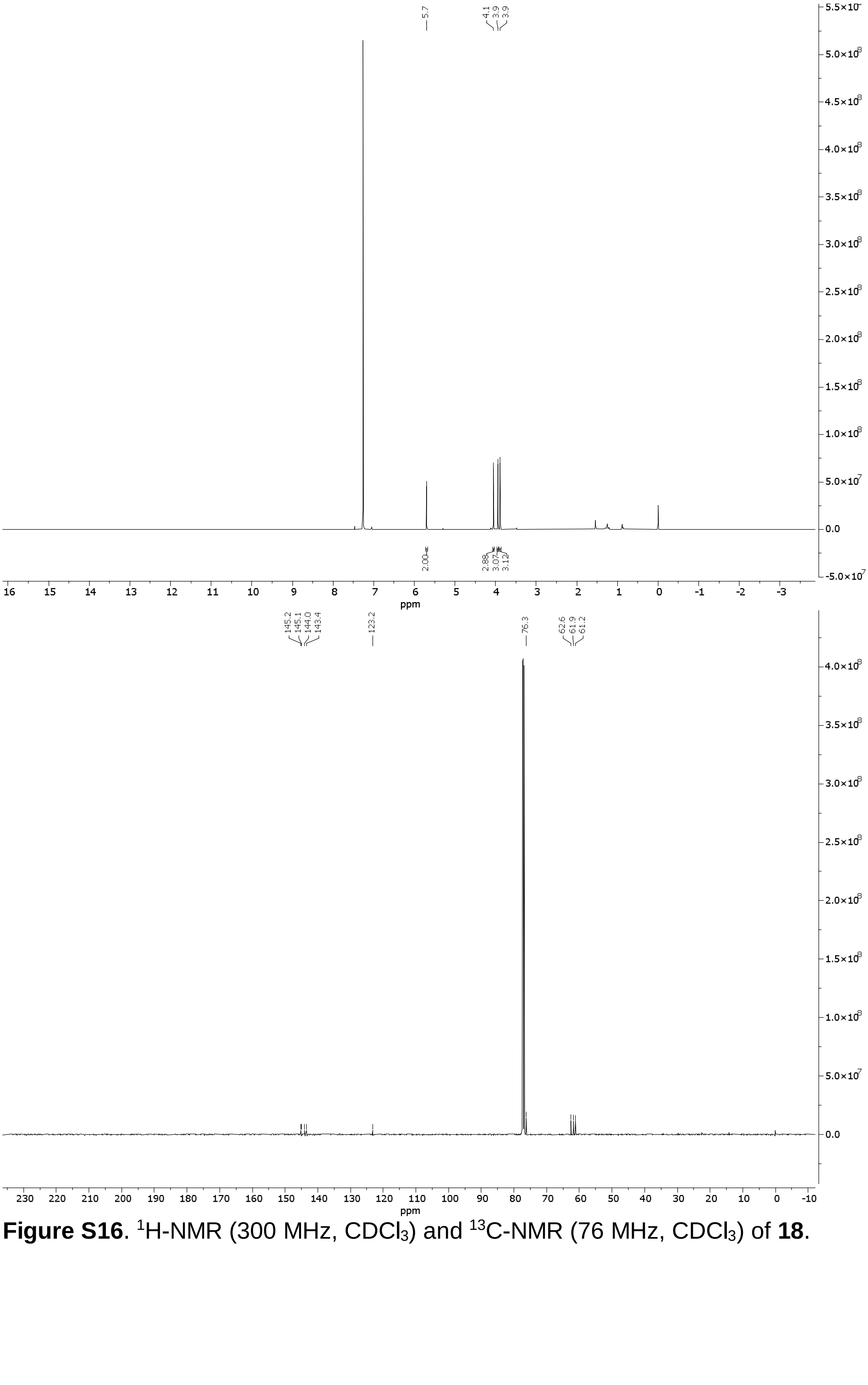

Figure S16. ^{1}H-NMR (300 MHz, $CDCl_3$) and ^{13}C-NMR (76 MHz, $CDCl_3$) of **18**.

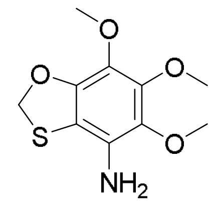

11

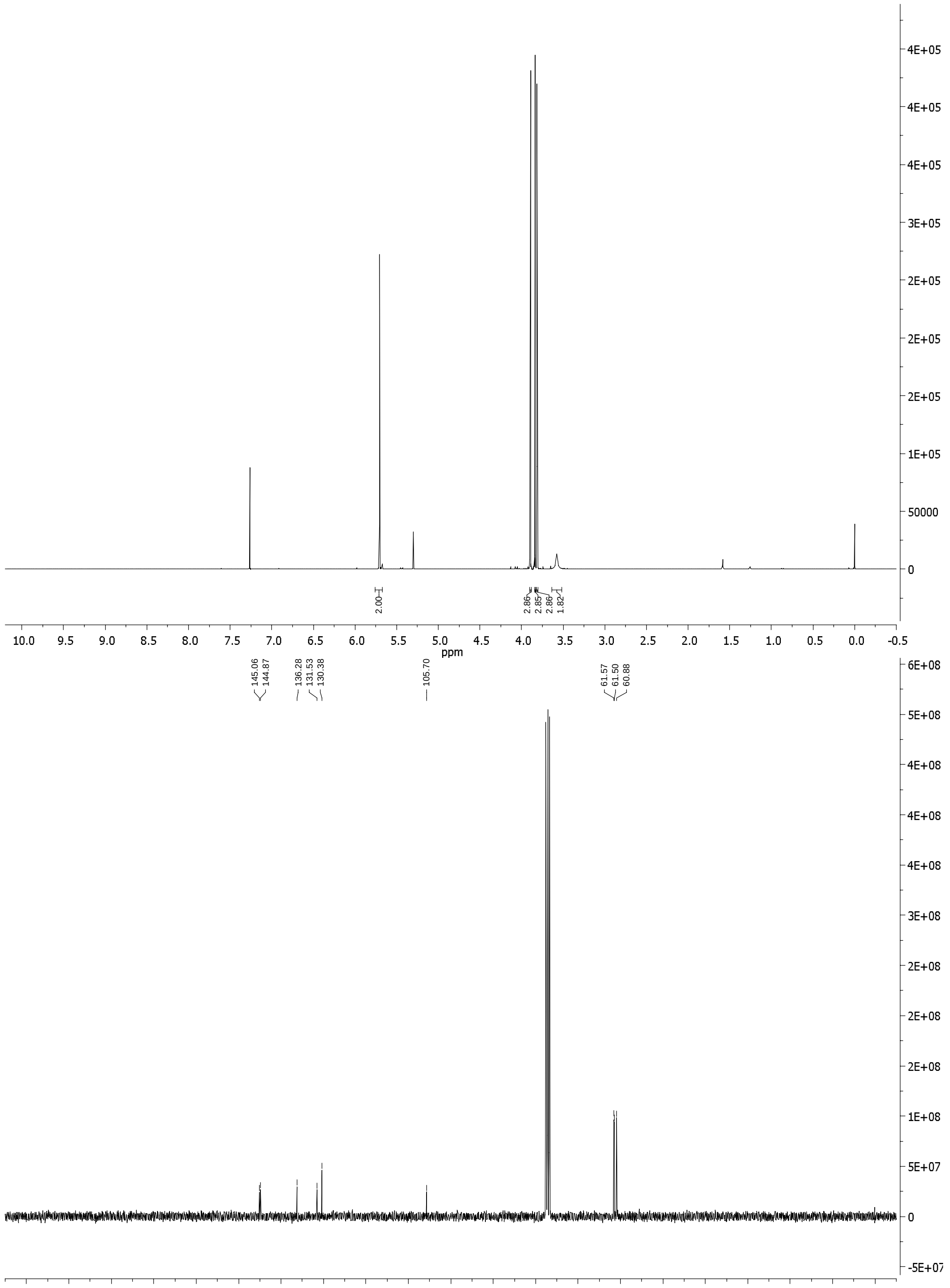

Figure S17. ^{1}H-NMR (300 MHz, $CDCl_3$) and ^{13}C-NMR (76 MHz, $CDCl_3$) of **11**.

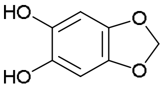

S26

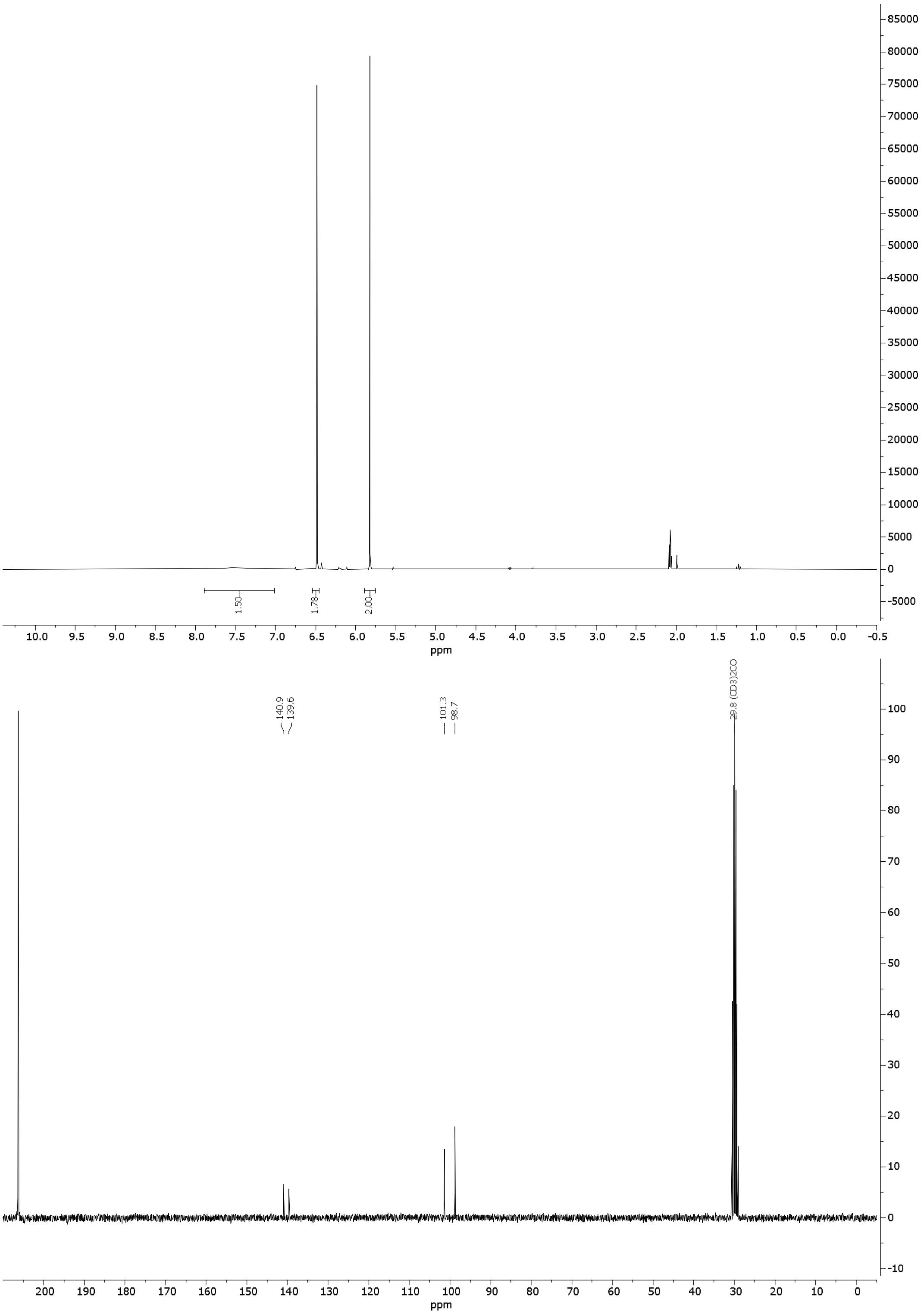

Figure S18. ^{1}H-NMR (300 MHz, $CDCl_3$) and ^{13}C-NMR (76 MHz, acetone) of **S27**.

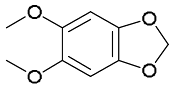

S27

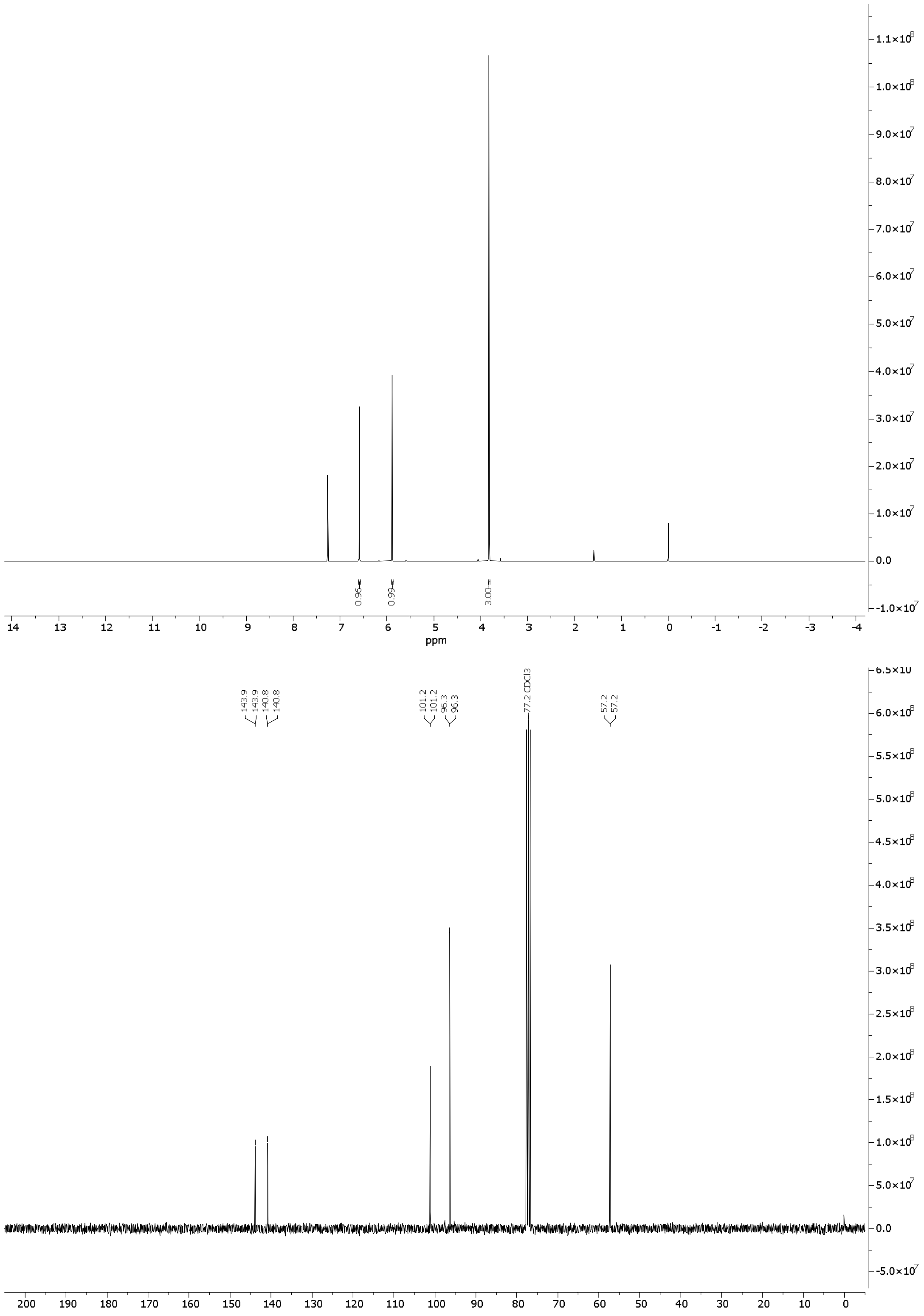

Figure S19. ^{1}H-NMR (300 MHz, $CDCl_3$) and ^{13}C-NMR (76 MHz, $CDCl_3$) of **S28**.

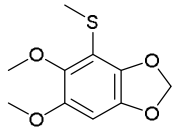

S12

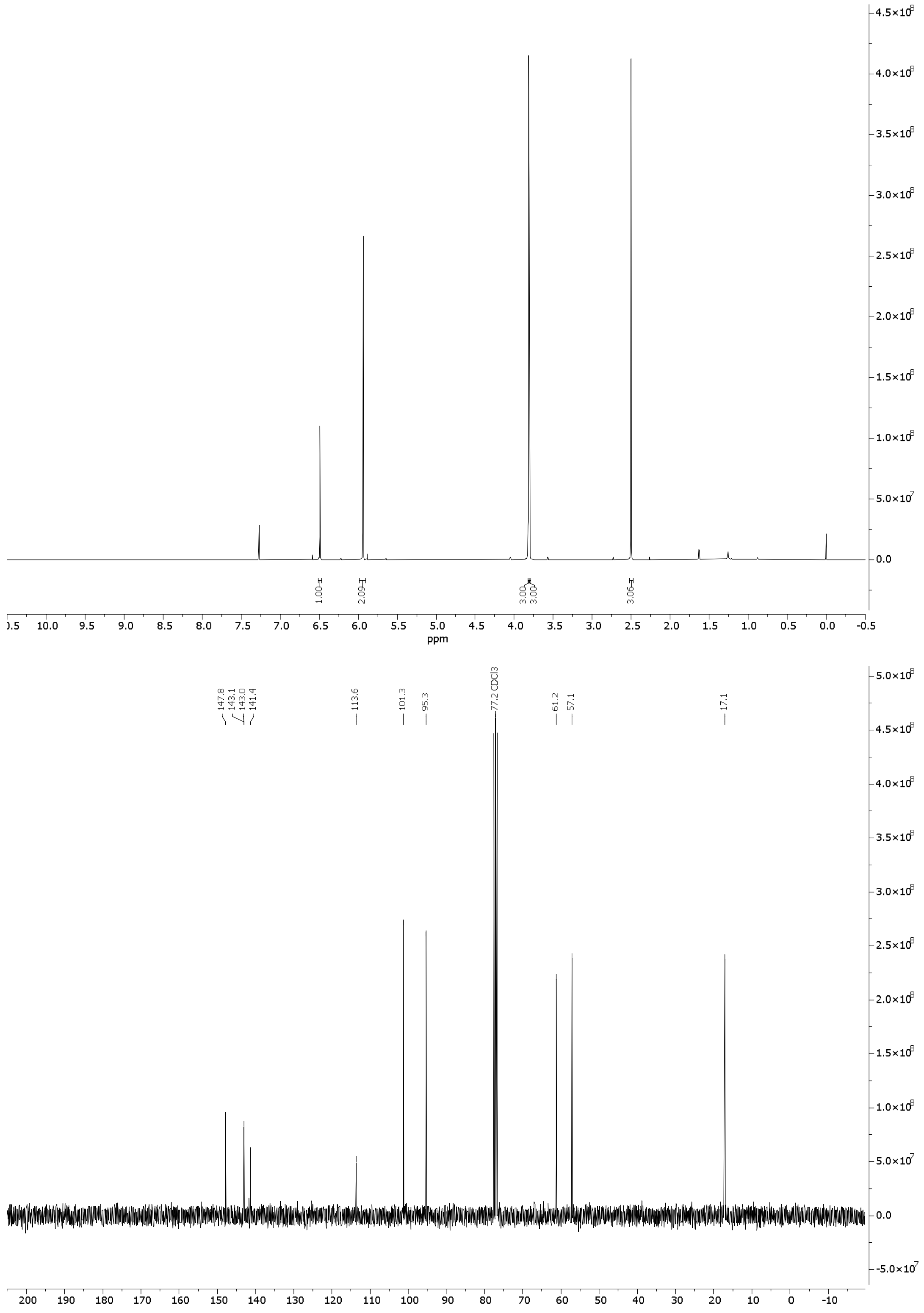

Figure S20. ^{1}H-NMR (300 MHz, $CDCl_3$) and ^{13}C-NMR (76 MHz, $CDCl_3$) of **S12**.

References

1 F. Pultar, M. E. Hansen, S. Wolfrum, L. Böselt, R. Fróis-Martins, S. Bloch, A. G. Kravina, D. Pehlivanoglu, C. Schäffer, S. LeibundGut-Landmann, S. Riniker and E. M. Carreira, Mutanobactin D from the Human Microbiome: Total Synthesis, Configurational Assignment, and Biological Evaluation, *J. Am. Chem. Soc.*, 2021, **143**, 10389–10402.

2 L. Böselt, R. Dötzer, S. Steiner, M. Stritzinger, S. Salzmann and S. Riniker, Determining the Regiochemistry and Relative Stereochemistry of Small and Druglike Molecules Using an Alignment Algorithm for Infrared Spectra, *Anal. Chem.*, 2020, **92**, 9124–9131.

3 H. van den Dool and P. Kratz, A generalization of the retention index system including linear temperature programmed gas-liquid partition chromatography, *J. Chromatogr.*, 1963, **11**, 463–471.

4 C. Schlawis, S. Kern, Y. Kudo, J. Grunenberg, B. S. Moore and S. Schulz, Structural Elucidation of Trace Components Combining GC/MS, GC/IR, DFT-Calculation and Synthesis—Salinilactones, Unprecedented Bicyclic Lactones from *Salinispora* Bacteria, *Angew. Chem. Int. Ed.*, 2018, **57**, 14921–14925.

5 T. A. Halgren, MMFF VII. Characterization of MMFF94, MMFF94s, and other widely available force fields for conformational energies and for intermolecular-interaction energies and geometries, *J. Comput. Chem.*, 1999, **20**, 730–748.

6 M. J. Frisch, G. W. Trucks, H. B. Schlegel, G. E. Scuseria, M. A. Robb, J. R. Cheeseman, G. Scalmani, V. Barone, G. A. Petersson, H. Nakatsuji, X. Li, M. Caricato, A. Marenich, J. Bloino, B. G. Janesko, R. Gomperts, B. Mennucci, H. P. Hratchian, J. V. Ortiz, A. F. Izmaylov, J. L. Sonnenberg, D. Williams-Young, F. Ding, F. Lipparini, F. Egidi, J. Goings, B. Peng, A. Petrone, T. Henderson, D. Ranasinghe, V. G. Zakrzewski, J. Gao, N. Rega, G. Zheng, W. Liang, M. Hada, M. Ehara, K. Toyota, R. Fukuda, J. Hasegawa, M. Ishida, T. Nakajima, Y. Honda, O. Kitao, H. Nakai, T. Vreven, K. Throssell, J. A. Montgomery, Jr., J. E. Peralta, F. Ogliaro, M. Bearpark, J. J. Heyd, E. Brothers, K. N. Kudin, V. N. Staroverov, T. Keith, R. Kobayashi, J. Normand, K. Raghavachari, A. Rendell, J. C. Burant, S. S. Iyengar, J. Tomasi, M. Cossi, J. M. Millam, M. Klene, C. Adamo, R. Cammi, J. W. Ochterski, R. L. Martin, K. Morokuma, O. Farkas, J. B. Foresman, D. J. Fox, Gaussian 09, *Gaussian, Inc., Wallingford CT*, **2009**.

7 A. D. Becke, Density-functional exchange-energy approximation with correct asymptotic behavior, *Phys. Rev. A*, 1988, **38**, 3098–3100.

8 J. E. Bello, P. Stamm, H. P. Leinaas and S. Schulz, Viaticene A – An Unusual Tetraterpene Cuticular Lipid Isolated from the Springtail *Hypogastrura viatica*, *Eur. J. Org. Chem.*, 2019, **2019**, 2158–2162.

9 W. Schmidt, T. M. Schulze, G. Brasse, E. Nagrodzka, M. Maczka, J. Zettel, P. G. Jones, J. Grunenberg, M. Hilker, U. Trauer-Kizilelma, U. Braun and S. Schulz, Sigillin A, a unique polychlorinated arthropod deterrent from the snow flea *Ceratophysella sigillata*, *Angew. Chem. Int. Ed.*, 2015, **54**, 7698–7702.

10 J. H. Rigby, U. S. M. Maharoof and M. E. Mateo, Studies on the Narciclasine Alkaloids: Total Synthesis of (+)-Narciclasine and (+)-Pancratistatin, *J. Am. Chem. Soc.*, 2000, **122**, 6624–6628.

11 *US Pat.*, US2014274701 (A1), 2014.

12 P. Kunyane, M. S. Sonopo and M. A. Selepe, Synthesis of Isoflavones by Tandem Demethylation and Ring-Opening/Cyclization of Methoxybenzoylbenzofurans, *J. Nat. Prod.*, 2019, **82**, 3074–3082.

13 M. Abbasi, N. Nowrouzi and H. Sedaghat, Efficient thiocyanation of aromatic compounds using NH_4SCN, DMSO and H_2SO_4, *Tetrahedron Lett.*, 2022, **101**, 153929.

14 Z. Lyu, Y. Zhao, Z. Y. Buuh, N. Gorman, A. R. Goldman, M. S. Islam, H.-Y. Tang and R. E. Wang, Steric-Free Bioorthogonal Labeling of Acetylation Substrates Based on a Fluorine-Thiol Displacement Reaction, *J. Am. Chem. Soc.*, 2021, **143**, 1341–1347.

15 *WO Pat.*, WO2023288013 (A2), 2022.
16 E. d. L. Chazin, P. d. S. Sanches, E. B. Lindgren, W. T. Vellasco Júnior, L. C. Pinto, R. M. R. Burbano, J. D. Yoneda, K. Z. Leal, C. R. B. Gomes, J. L. Wardell, S. M. S. V. Wardell, R. C. Montenegro and T. R. A. Vasconcelos, Synthesis and biological evaluation of novel 6-hydroxy-benzo[d][1,3]oxathiol-2-one Schiff bases as potential anticancer agents, *Molecules*, 2015, **20**, 1968–1983.
17 M. K. Prashanth, H. D. Revanasiddappa, K. M. Lokanatha Rai and B. Veeresh, Synthesis, characterization, antidepressant and antioxidant activity of novel piperamides bearing piperidine and piperazine analogues, *Bioorg. Med. Chem. Lett.*, 2012, **22**, 7065–7070.
